# Correlation between evoked neurotransmitter release and adaptive functions in SYT1-associated neurodevelopmental disorder

**DOI:** 10.1016/j.ebiom.2024.105416

**Published:** 2024-10-30

**Authors:** Paul Yangho Park, Lauren Elizabeth Bleakley, Nadia Saraya, Reem Al-Jawahiri, Josefine Eck, Marc Anthony Aloi, Holly Melland, Kate Baker, Sarah Louise Gordon

**Affiliations:** a The Florey Institute of Neuroscience and Mental Health, University of Melbourne, Parkville, 3052, VIC, Australia; b MRC Cognition and Brain Sciences Unit, University of Cambridge, Cambridge, CB2 7EF, UK; c Drug Discovery Biology, Monash Institute of Pharmaceutical Sciences, Faculty of Pharmacy and Pharmaceutical Sciences, Monash University, Parkville, 3052, VIC, Australia; d Neuromedicines Discovery Centre, Monash University, Parkville, 3052, VIC, Australia; e Department of Medical Genetics, University of Cambridge, Cambridge, CB2 0XY, UK

**Keywords:** Synaptotagmin, Neurodevelopmental disorder, Intellectual disability, Exocytosis, Neurotransmission, Motor delay

## Abstract

**Background:**

Pathogenic missense variants in the essential synaptic vesicle protein synaptotagmin-1 (SYT1) cause a neurodevelopmental disorder characterised by motor delay and intellectual disability, hyperkinetic movement disorder, episodic agitation, and visual impairments. SYT1 is the presynaptic calcium sensor that triggers synchronous neurotransmitter release. We have previously shown that pathogenic variants around the calcium-sensing region of the critical C2B domain decrease synaptic vesicle exocytosis in neurons.

**Methods:**

Here, we have used cultured hippocampal neurons transfected with SYT1-pHluorin to examine how variants within the C2A and C2B domain of SYT1 impact evoked exocytosis.

**Findings:**

We show that recently identified variants within the facilitatory C2A domain of the protein (L159R, T196K, E209K, E219Q), as well as additional variants in the C2B domain (M303V, S309P, Y365C, G369D), share an underlying pathogenic mechanism, causing a graded and variant-dependent dominant-negative impairment in exocytosis. We establish that the extent of evoked exocytosis observed *in vitro* in the presence of SYT1 variants correlates with neurodevelopmental impacts of this disorder. Specifically, the severity of motor and communication impairments exhibited by individuals harbouring these variants correlates with multiple measures of exocytic efficiency.

**Interpretation:**

Together, this suggests that there is a genotype-function-phenotype relationship in SYT1-associated neurodevelopmental disorder, centring impaired evoked neurotransmitter release as a common pathogenic driver. Moreover, this points toward a direct link between control of neurotransmitter release and development of adaptive functions, providing a tractable target for therapeutic amelioration.

**Funding:**

Australian National Health and Medical Research Council, 10.13039/501100000265UK Medical Research Council, 10.13039/501100001279Great Ormond Street Hospital Children's Charity, 10.13039/501100001782University of Melbourne.

**Research in context**

**Evidence before this study**

Genetic variants in synaptotagmin-1 (SYT1), an essential calcium sensor that triggers action potential-evoked neurotransmitter release, cause a neurodevelopmental disorder. *In vitro* studies revealed that variants in the C2B domain of the protein induced dominant-negative impairment of synaptic vesicle exocytosis. Recently, additional neurodevelopmental disorder-associated SYT1 variants have been identified, both within the critical C2B domain as well as the facilitatory C2A domain of the protein, but a pathogenic mechanism for these variants is yet to be established.

**Added value of this study**

In this current study, we first aimed to identify impairments to neuronal physiology induced by neurodevelopmental disorder-associated variants in the C2A and C2B domains of SYT1, in order to establish their pathogenicity and identify potential diversity of functional impacts. We revealed that variants in both the C2A and C2B domain of SYT1 induce a dominant-negative impairment to evoked exocytosis but do so in a graded, variant-specific manner. Secondly, we interrogated whether the observed functional impacts contribute to variable severity of the associated neurodevelopmental condition, demonstrating that the efficiency of evoked exocytosis in the presence of SYT1 variants correlates with the severity of motor and communication impairments exhibited by individuals harbouring these variants.

**Implications of all the available evidence**

Together, this suggests a direct link between control of neurotransmitter release and development of adaptive functions. With the finding that perturbed exocytosis is common to both C2A and C2B SYT1 variants, and that this correlates with disorder severity, impairments to evoked neurotransmitter release emerge as a core tractable target to investigate for therapeutic amelioration of SYT1-associated neurodevelopmental disorder.

## Introduction

High-fidelity neurotransmission is achieved through tight coupling of action potentials (AP) at nerve terminals to synaptic vesicle exocytosis. In the cerebrum, this is primarily mediated by the presynaptic vesicular calcium sensor synaptotagmin-1 (SYT1).^1^ SYT1 inhibits vesicle fusion in resting neurons^2^, ^3^, ^4^; upon the influx of calcium through voltage-gated calcium channels, calcium binds to SYT1, acting as an electrostatic switch allowing the protein to penetrate the plasma membrane.^5^, ^6^, ^7^, ^8^, ^9^ This triggers and facilitates the fusion of neurotransmitter-laden synaptic vesicles with the presynaptic plasma membrane,^10^ and thus the release of neurotransmitters into the synaptic cleft. Complete loss of SYT1 results in early lethality and a profound abrogation of evoked neurotransmitter release in excitatory and inhibitory neurons with a concomitant increase in both spontaneous and asynchronous release.^11^, ^12^, ^13^

The two calcium-binding C2A and C2B domains of SYT1 cooperate in membrane binding and vesicle fusion. Calcium binding to the C2B domain is essential for triggering synchronous evoked release.^7^^,^^14^ This domain interacts with the pre-fusion soluble N-ethylmaleimide-sensitive factor activating protein receptor (SNARE) complex to effectively position SYT1 to respond to calcium influx, and is an energetic driver of membrane fusion.^15^^,^^16^ In comparison, the C2A domain plays an important facilitatory role in vesicle fusion by helping activate the electrostatic switch that triggers synchronous release,^17^^,^^18^ while also demonstrating functional cooperativity with the C2B domain.^16^^,^^19^ The lack of a functional C2A domain results in a significant reduction, though not complete ablation, of evoked neurotransmitter release.^9^^,^^18^, ^19^, ^20^ Dysfunctional SYT1, caused by strategic mutations to either C2 domain, perturbs both excitatory and inhibitory neurotransmission in Drosophila, or when introduced into murine SYT1 knockout neurons.^21^, ^22^, ^23^, ^24^ Furthermore, disruption to normal SYT1 function in mammalian systems can significantly impair both short-term^25^ and long-term^24^ synaptic plasticity, as well as altering synaptic short-term depression.^26^ This highlights the relevance of SYT1 and neurotransmission kinetics for the synaptic correlates of learning and cognition. However, studies investigating behavioural impacts of SYT1 variants in mammalian model systems remain limited.^11^^,^^27^

Heterozygous *de novo* missense variants in *SYT1* cause a rare condition known as SYT1-associated neurodevelopmental disorder (OMIM #618218, Baker–Gordon Syndrome or BAGOS).^28^^,^^29^ Frequently reported clinical features of this disorder include infantile hypotonia, congenital ophthalmic abnormalities, early-onset hyperkinetic movement disorders, and developmental delay/intellectual disabilities.^28^, ^29^, ^30^ A characteristic feature of the condition is electroencephalogram (EEG) abnormalities (most often low frequency, high amplitude oscillations), which are not accompanied by overt seizures or abnormal structural neuroimaging in most cases.^28^^,^^29^

The first human pathogenic variants identified in SYT1 consisted of single amino acid substitutions in residues within the Ca^2+^-binding loops of the C2B domain of the protein.^28^^,^^29^ *In vitro* studies revealed that, while one variant (M303K) caused a reduction in protein stability and expression, all other variants (D304G, D366E, I368T and N371K) induced dominant-negative impairment of synaptic vesicle exocytosis.^28^^,^^29^^,^^31^ These findings provided the first evidence of a pathogenic mechanism underlying the condition. This disruption to exocytosis was demonstrated to be reversible by increasing intracellular calcium concentrations, either via application of increased extracellular calcium or the potassium-channel blocker 4-aminopyridine (4-AP).^29^^,^^31^

Recently, additional disorder-associated SYT1 variants have been identified, both within the C2B domain (including outside of the calcium-binding pocket) and within the C2A domain of the protein.^30^^,^^32^^,^^33^ Molecular dynamics simulations demonstrated minimal evidence of structural abnormalities being a major component to the mechanisms of pathogenicity for most of these variants,^30^ with a pathogenic mechanism for these emerging variants yet to be established. In parallel with the increasing number and diversity of reported SYT1 variants, standardised phenotyping revealed a broadened range in the severity of associated neurodevelopmental characteristics.^30^ The extent of developmental delay and intellectual disability varied from mild to profound; 66% of 22 patients were reported to have a movement disorder, ranging from isolated ataxia or dystonia to severe mixed hyperkinetic involuntary symptoms.^30^ Other common features included visual impairments, emotional instability and self-injurious behaviours, which occurred at higher frequency than observed in a comparison group with other monogenic neurodevelopmental disorders, but varied widely in severity within the SYT1 group. A simple dichotomy in the severity of symptoms could not be found between individuals with C2A or C2B domain variants, suggesting that a more complex genotype-specific impact on SYT1 function might influence phenotypic severity.

Building on these previous observations, the current study had two major objectives. Firstly, we aimed to identify impairments to neuronal physiology induced by emerging variants in the C2A and C2B domains of SYT1, in order to establish their pathogenicity and identify potential diversity of functional impacts. We specifically investigated missense variants across the two domains that were predicted to have differential impacts on SYT1 structure and function.^30^ Secondly, we interrogated whether the observed functional impacts contribute to variable severity of the associated neurodevelopmental condition. The recurrent C2B variant I368T has well-characterised functional impacts, and hence was included as a reference variant.^28^^,^^29^^,^^34^^[preprint]^ We reveal here a shared mechanism of pathogenicity that correlates with core neurodevelopmental features of SYT1-associated neurodevelopmental disorder.

## Methods

### Ethics

This study was conducted within the “Phenotypes in Intellectual Disability” project, which received approval from the Cambridge Central Research Ethics Committee (REC ref: IRAS 83633). Written informed consent was provided by each diagnosed individual's parent or consultee prior to information sharing by referring clinicians and questionnaire completion by the parents or carers. All animal experiments were performed in accordance with the Prevention of Cruelty to Animals Act, 1986 under the guidelines of the National Health and Medical Research Council (NHMRC) of Australia Code of Practice for the Care and Use of Animals for Experimental Purposes. All experiments were approved by the Animal Ethics Committee at the Florey Institute of Neuroscience and Mental Health (project number 20–009 and 22-024) prior to commencement.

### Materials

SYT1-pHluorin (a pH-sensitive variant of GFP) in a modified pcDNA3 vector carrying a neuron-specific synapsin promoter^35^ was provided by Prof. V. Haucke (Leibniz Institute of Molecular Pharmacology, Berlin, Germany). QuikChange II Site-Directed Mutagenesis kit was from Agilent Technologies (Cat#: 200523, Santa Clara, CA, USA). Thermo Plasmid Maxiprep Kit (Cat#: K210017), Neurobasal media (Cat#: 21103049), B-27 supplement (Cat#: 17504044), penicillin/streptomycin (Cat#: 15140122), Minimal Essential Medium (MEM) (Cat#: 11095080), Dulbecco's MEM/F-12 (Cat#: 11320033), Lipofectamine 2000 (Cat#: 11668019), goat anti-chicken IgY (H + L) Alexa Fluor 488 (Cat#: A11039, RRID: AB_2534096) and donkey anti-rabbit IgG (H + L) DyLight 550 (Cat#: SA5-10039, RRID: AB_2556619) were obtained from ThermoFisher Scientific (Scoresby, Australia). Rabbit anti-SYT1 was from Synaptic Systems (Cat#: 105102, RRID: AB_887835, Göttingen, Germany) and ThermoFisher Scientific (Cat#: PA5-87551, RRID: AB_2804245, Scoresby, Australia). Chicken anti-GFP was from Abcam (Cat#: ab13970, RRID: AB_300798, Cambridge, UK). 6-cyano-7-nitroquinoxaline-2,3-dione (CNQX) was from ENZO Life Sciences (Cat#: A16110, Lausen, Switzerland). DL-2-Amino-5-phosphonopentanoic acid (DL-AP5) (Cat#: 14540) and Bafilomycin A1 (Cat#: 11038) were from Cayman Chemical (Ann Arbor, MI, USA). All other reagents were obtained from Sigma–Aldrich (Castle Hill, Australia).

### Functional studies

#### Site-directed mutagenesis

Site-directed mutagenesis was used to introduce the human SYT1 variants into homologous positions in rat SYT1 (human/rat: L159/158>R, T196/195>K, E209/208>K, E219/218>Q, M303/302>V, S309/S308>P, Y365/364>C, G369/368>D, I368/367>T, amino acid numbering used henceforth follows the human sequence), that was fused at the N-terminal lumenal domain via a 25 amino acid spacer to superecliptic pHluorin, a pH-sensitive enhanced green fluorescent protein (EGFP).^36^ I368T SYT1-pHluorin was previously generated as described.^28^ The DNA sequences of human and rat SYT1 demonstrate 97% similarity overall, and are 100% homologous in their C2A and C2B domains.^37^ Mutagenesis of SYT1-pHluorin was performed using the QuickChange II kit (Agilent) using primers listed in Supplementary Table S1, and successful mutagenesis of plasmid DNA was confirmed through Sanger sequencing performed by the Australian Genome Research Facility, Melbourne, Australia. Transfection-grade plasmids were produced using the Thermo Plasmid Maxiprep Kit as per the manufacturer's instructions.

#### Primary hippocampal neuronal culture

C57BL/6J mouse in-house colonies were maintained in a temperature controlled (≈21 °C) room and group housed in individually ventilated cages on a 12 h light–dark cycle (lights on 07:00–19:00) with food and water available ad libitum, and nesting material and housing boxes for environmental enrichment. Animals were time mated overnight and visualisation of a vaginal plug on the following morning was considered as embryonic day (E) 0.5.

Dissociated primary hippocampal-enriched neuronal cultures were prepared from hippocampi dissected from at least three E16.5–18.5 C57BL/6J mouse embryos of both sexes. Hippocampi were incubated in 10 units/mL papain for 20 min at 37 °C; papain was removed, and the hippocampal tissue was mechanically disaggregated in pre-equilibrated DMEM/F-12 media supplemented with 10% foetal bovine serum and 1% penicillin/streptomycin. The cell suspension was centrifuged at 363 g for 5 min, and the hippocampal cell pellet was resuspended in fresh, pre-equilibrated Neurobasal (NB) media supplemented with 1% penicillin-streptomycin, 0.5 mM l-glutamine and 1x B-27 (henceforth termed “enriched NB”). Harvested cells were subsequently plated onto poly-d-lysine and laminin-coated coverslips at a density of 35,000 cells/coverslip for 24-well plates (on 13 mm coverslips) and 60,000 cells/coverslip for 6-well plates (on 25 mm coverslips), after which they were incubated for an hour at 37 °C, 5% CO_2_ before supplementation with pre-equilibrated enriched NB media. At 3-4 days *in vitro* (i.e. DIV3-4), cultures were further supplemented with 1 μM 1-β-d-arabinofuranosylcytosine to inhibit glial proliferation.

#### Transfection

At DIV6-7, the hippocampal neuronal cultures were co-transfected with SYT1-pHluorin variants in the presence of either pcDNA empty vector for fixed cell imaging, or mCherry for live cell imaging assays. Lipofectamine 2000 (1 μL/well for 24-well plates, 2 μL/well for 6-well plates) was incubated in MEM (125 μL/well for 24-well plates, 250 μL/well for 6-well plates) for 5 min at room temperature. Plasmid DNA (0.5 μg/construct/well for 24-well plates, 1 μg/construct/well for 6-well plates) in MEM media (125 μL/well for 24-well plates, 250 μL/well for 6-well plates) was combined with an equal volume of lipofectamine mixture and incubated at room temperature for 20 min. Enriched NB media was collected from cells and retained, and cells were transfected in pre-equilibrated MEM for 2 h at 37 °C, 5% CO_2_. The cells were gently washed with MEM and the conditioned enriched NB media replaced, and cells maintained at 37 °C, 5% CO_2_ until use. This results in a transfection efficiency of approximately 5% of neurons.

#### Fixation & immunocytochemistry

At DIV13 transfected primary hippocampal neuronal cultures were first washed with saline imaging buffer (in mM: 136 NaCl, 2.5 KCl, 2 CaCl_2_, 1.3 MgCl_2_, 10 glucose, 10 HEPES, pH 7.4) and then fixed in 4% paraformaldehyde in phosphate-buffered saline (PBS) for 20 min, incubated at room temperature in 50 mM NH_4_Cl in PBS for 10 min, washed with PBS and permeabilised with 0.1% v/v Triton x100, 1% v/v bovine serum albumin (BSA) in PBS for 5 min. Cells were then washed with PBS and blocked with 1% BSA in PBS for 1 h. Cells were subsequently immunolabelled with primary antibodies in 1% BSA in PBS (1:2000 chicken anti-GFP, Abcam, Cat#: ab13970; 1:100 rabbit anti-SYT1, Synaptic Systems, Cat#: 105102 or 1:200 rabbit anti-SYT1, ThermoFisher Scientific, Cat#: PA5-87551) in a dark humidified chamber for 1.5–2 h, washed extensively with PBS, then immunolabelled in the dark with secondary antibodies in 1% BSA in PBS (1:500 488 Alexafluor goat anti-chicken, Cat#: A11039; 1:200 550 DyLight donkey anti-rabbit, Cat#: SA5-10039, both ThermoFisher Scientific) over 1 h. Finally, all cells were labelled with 4’,6-diamidino-2-phenylindole (DAPI) for maximum of 5 min. Cells were washed 3 times with PBS after each incubation period to remove excess antibodies. Coverslips were then rinsed twice in fresh double-distilled H_2_O, then mounted on glass slides using Dako Mounting Medium.

#### Fluorescence imaging

Fixed, immunolabelled cells or live neuronal cultures were imaged on a Zeiss Axio Observer 7 inverted epifluorescence microscope with a Colibri 7 LED light source. Live neuronal cultures on 25 mm coverslips were mounted in a Warner imaging chamber with embedded parallel platinum wires (RC-21BRFS) before being placed on the stage of the microscope. All neurons were visualised through a Zeiss EC Plan-Neofluar 40x/1.30 oil-immersion objective and a Zeiss Axiocam 506 mono camera. Single focal plane 14-bit images were captured with 2 × 2 binning yielding a pixel size of 0.227 μm.

SYT1-pHluorin was visualised with a 488 nm excitation wavelength through a single bandpass GFP filter set (excitation 470/40, emission 525/50, beam splitter 495 nm). SYT1 and cell soma were visualised through AF555 and DAPI channels respectively, with excitation wavelengths of 553 and 353 nm respectively through a quadruple bandpass 90 HE filter set (excitation 385/20, 469/28, 555/20, 632/24 and emission 425/30, 514/30, 591/25, 709/100, beam splitter 405/493/575/653 nm). mCherry was visualised with a 545 nm excitation wavelength through a single bandpass DsRed filter set (excitation 550/24, emission 605/70, beam splitter 570 nm).

For live imaging, all buffers were warmed to 37 °C via a Warner inline heater, and neurons were stimulated via a D343 Dual Stimulator module employed in a D330 MultiStim System (SDR Scientific Pty Ltd, Chatswood, Australia). Automated triggering of electrical stimulation and variable image acquisition rates were implemented using the Zen Blue Experiment Designer module.

For the membrane partitioning assay, live hippocampal neuronal cultures were perfused sequentially with saline imaging buffer (as described above), 20 mM 2-ethanesulfonic acid (MES) buffer (in mM: 136 NaCl, 2.5 KCl, 2 CaCl_2_, 1.3 MgCl_2_, 10 glucose, 20 MES, pH 5.5), and 50 mM NH_4_Cl alkaline imaging buffer (in mM: 86 NaCl, 2.5 KCl, 2 CaCl_2_, 1.3 MgCl_2_, 10 glucose, 10 HEPES, 50 NH_4_Cl, pH 7.4). For the exocytosis assay, live cultures were perfused with saline imaging buffer (as described above, supplemented with 10 μM CNQX, 50 μM DL-AP5 and 1 μM vATPase inhibitor bafilomycin A1) at 37 °C. Neurons were stimulated with either 1200 AP at 10 Hz (35 mA, 1 ms pulse width) for 2 min, or stimulated with 40 AP at 20 Hz for 2 s followed by a 3 s recovery, then at 10 Hz for 2 min. Neurons were then perfused with an alkaline NH_4_Cl buffer (as described above) to reveal the total pHluorin fluorescence. Images were captured at 1s intervals, except under the 5 s of 20 Hz stimulation and recovery, where they were acquired at 10 Hz frequency.

#### Imaging analysis

Images were analysed using the FIJI 1.52n distribution of ImageJ and the Time Series Analyzer V3 plugin. Nerve terminal SYT1 protein level analysis of fixed cell images were carried out by first selecting 8 × 8 pixel regions of interest (ROIs) over transfected (identified via pHluorin immunolabelling) and non-transfected nerve terminals and background regions. For somatic SYT1 expression, five 10 x 10 pixel ROIs were placed over transfected (identified via pHluorin immunolabelling) and non-transfected cell soma, as well as background regions. SYT1 levels in transfected neurons was calculated using Microsoft Excel as fold-change over the non-transfected endogenous SYT1 levels by dividing the average fluorescence intensity of transfected puncta/soma by that of non-transfected puncta/soma after subtracting background intensity.

To correct for slight XY-plane drift over the time series, stack registration was performed by rigid body alignment to the first frame of the experiment using the MBGReg ImageJ plugin (Donal Stewart, Edinburgh, UK), a custom variant of the FIJI StackReg plugin. For live cell imaging analysis, 10 × 10 pixel ROIs were selected over transfected nerve terminals (identified by fluorescence response to NH_4_Cl buffer) as well as background. To remove the component of fluorescence decay contributed by the photobleaching of background autofluorescence in time series images from evoked exocytosis assays, the Bleach Correction Image J plugin was used (version: CorrectBleach_-2.0.3-SNAPSHOT.jar, https://github.com/fiji/CorrectBleach).^38^ Photobleaching over the full length of the experiment was corrected using a single exponential function fitted to the fluorescence decay of background ROIs over the duration of the experiment prior to NH_4_Cl buffer superfusion. After bleach-correcting against the selected set of background ROIs, transfected ROIs were laid over the image and synaptic fluorescence intensities measured. Each transfected ROI subsequently underwent a rigorous screening process with strict inclusion/exclusion criteria. Only ROIs that increased fluorescence from baseline upon both electrical stimulation and NH_4_Cl buffer superfusion were included in subsequent analysis, and coverslips with greater than 20 responsive ROIs were included in the final data.

For exocytosis assays, fluorescence responses of each ROI were calculated in Microsoft Excel as ΔF/F_0_ (change in fluorescence over baseline where baseline is the average fluorescence of the 5 frames before stimulation). For each frame of the time series, ΔF/F_0_ fluorescence values were averaged for all ROIs in a field of view, then baseline (i.e 1) subtracted. The average fluorescence intensity traces were subsequently normalised through one of two methods; fluorescence intensities were either normalised to the peak fluorescence at the end of stimulation, or to the peak fluorescence exhibited in response to the NH_4_Cl alkaline buffer.

The diffuseness of SYT1-pHluorin fluorescence along axons and relative enrichment at nerve terminals was determined by calculating the coefficient of variation (CV) as previously described,^28^^,^^29^^,^^39^^,^^40^ using images from NH_4_Cl buffer-perfused cultures acquired during the membrane partitioning assay. In brief, single pixel lines >60 μm long were drawn over lengths of neurite, and CV of fluorescence measured (standard deviation of fluorescence/mean fluorescence). ‘n’ refers to the mean of 5 different >60 μm axonal segments from a single field of view.

### Participant recruitment and phenotyping

*SYT1* variants were identified via exome sequencing (trio or solo) within clinical laboratories or ethically approved research studies as previously reported.^29^^,^^30^ The identification, validation, confirmation of *de novo* status and clinical reporting of *SYT1* variants were carried out by each participant's clinical centre. Authors were notified of diagnosed variants by personal communication, through database searching of ClinVar,^41^ Decipher,^42^ or through Gene-Matcher.^43^ Written informed consent for inclusion in the current study was provided by each diagnosed individual's parent or consultee (Cambridge Central Research Ethics Committee ref IRAS 83633: “Phenotypes in Intellectual Disability”). Clinical information was collated from medical documentation. Parent-report questionnaires were completed online or by post or by online interview as reported.^30^ The study-specific Medical History Interview gathered information about perinatal history, infant and child health, neurological symptoms, and developmental milestones. Questionnaires or interviews were administered and scored by graduate psychologists, overseen by a qualified paediatrician. Given the rare nature of SYT1 variants, biological sex was not taken into account for study design; nevertheless, there is an equal number of male and female participants in this study. Biological sex was reported by parents/carers. The number of reported movement disorder types was summed into an ordinal score. The Vineland Adaptive Behaviour Scales (third edition, VABS)^44^ is a standardised assessment tool for everyday adaptive functioning domains (communication, daily living skills, socialisation, and motor skills). The Developmental Behaviour Checklist 2 (DBC)^45^ assesses emotional and behavioural problems in individuals with neurodevelopmental disorders and comprises 5 subscales (disruptive, self-absorbed, communication disturbance, anxiety and social relating). In line with our previous work, analysis focused on five items reflecting frequently reported symptoms within the *SYT1* group (6. Bangs head; 10. Chews or mouths body parts; 33. Hits or bites self; 47. Mood changes rapidly for no reason; 60. Has repeated movements of hands, body or head). These items appear across two DBC-P subscales (disruptive/antisocial, self-absorbed). Raw scores (0-2) for each item were summed for each participant. The Cerebral Visual Impairment (CVI) scale^46^ comprises six subscales (visual attitude, ventral stream, dorsal stream, complex visuomotor abilities, other senses, and associated characteristics), and analysis focused here on total scores. *SYT1* group data is outlined in Supplementary Tables S3 and S4.

### Statistics

#### Statistical analyses of functional data

Statistical analyses of SYT1 variant functional data were performed using GraphPad Prism Version 9.4.0 and Microsoft® Excel software. Q–Q residual plots were generated to assess normality; each data set was also formally tested for normality using the Shapiro–Wilk test and statistical analyses were performed accordingly (one-way ANOVA with Dunnett's multiple comparison test for data sets with normal distribution, non-parametric Kruskal–Wallis test with Dunn's multiple comparisons for data sets that did not conform to normal distribution, with each SYT1 variant protein compared to the WT protein). Full details, including Brown–Forsythe test for homoscedasticity, are reported in Supplementary Figure S4 and Supplementary Materials. Time courses of change in fluorescence were analysed via repeated measures mixed model ANOVA with no assumption of sphericity and using the Geisser-Greenhouse correction, with Dunnett's multiple comparison test, to compare each SYT1 variant to the WT control at every individual time point. Assumptions for repeated measures ANOVA were assessed and validated through Q–Q residual plots (for normality) and homoscedasticity plots (for homogeneity of variance) (Supplementary Figure S5). For all analyses, p < 0.05 was considered statistically significant where the degree of significance is represented by: ∗p < 0.05, ∗∗p < 0.01, ∗∗∗p < 0.001, ∗∗∗∗p < 0.0001. All data are displayed as mean ± SEM, where individual data points are shown as diamonds. ‘n’ refers to an individual field of view from an independent coverslip. Sample numbers are indicated in the graphs as single datapoints, and in the figure legends. No data that met inclusion criterion has been excluded from this study. Experimenters were blinded to the SYT1-pHluorin variant for image acquisition and analysis for fixed assays, and at the image analysis stage for live imaging. A study protocol was not pre-determined prior to the onset of the study; instead, experiments were based on outcomes of prior work. No strategy for randomization of samples has been followed, and sample sizes were not chosen based on pre-specified effect sizes. Instead, several independent experiments were performed across multiple independent cultures as indicated in figure legends, with all experiments repeated across at least 3 independent cultures (with each culture comprising at least 3 embryos). To minimise confounds, controls (WT) were performed in each batch of experiments alongside SYT1 variants, and the well-characterised recurrent C2B variant I368T was included as a reference variant in each assay.

#### Statistical analyses of functional and clinical data correlations

Permutation-based Spearman's correlation tests were performed to examine associations between functional activity (i.e. measures of *in vitro* synaptic vesicle exocytosis kinetics) and quantitative phenotyping measures for participants with the equivalent SYT1 variants. The SYT1 variants included in this analysis, based on available functional and phenotypic data, are the following: T196K, E209K, E219Q, M303V, S309P (DBC data not available), Y365C, G369D (all variants n = 1 participants), and I368T (n = 6 participants, mean scores assessed). To account for multiple comparisons, the false discovery rate was controlled using the Benjamini-Hochberg procedure, with q value < 0.05 (p ≤ 0.019). Correlations with CVI total score and DBC self-injury items score controlled for age. For other measures, bivariate correlation tests were appropriate as the variables represented standardised scores accounting for age.

## Role of funders

This work was supported by National Health and Medical Research Council (NHMRC) Ideas Grant (2003710) to SG, UK Medical Research Council (MC_UU_00030/3) and Great Ormond Street Hospital Children's Charity to KB, and a University of Melbourne Early Career Researcher Grant to LEB. PYP acknowledges the support of an Australian Government Research Training Program Scholarship. No funders had any role in conducting the actual study, including the study design, data collection, data analyses, interpretation and writing of reports.

## Results

### Most SYT1 variants do not impact SYT1 expression or trafficking

**Fig. 1:**
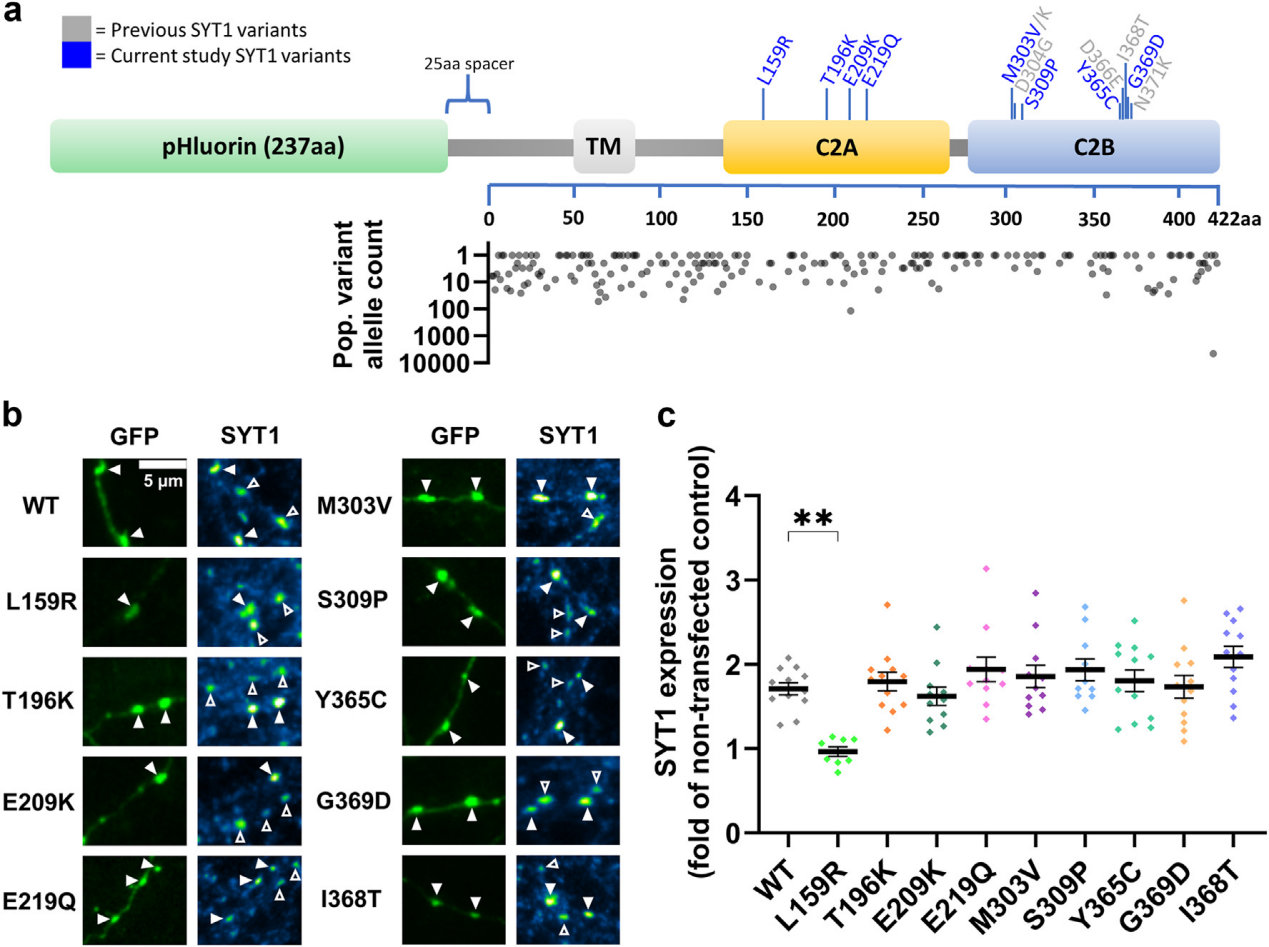
**SYT1 variants, except L159R, are expressed as efficiently as the WT protein at presynaptic nerve terminals.** Hippocampal neurons co-transfected with SYT1-pHluorin variants and empty vector pcDNA3.1 were fixed and immunolabelled for GFP (i.e. pHluorin, to identify transfected neurons) and SYT1. (**a**) Linear protein sequence of SYT1-pHluorin construct highlighting the location of previously investigated (grey) and currently examined (blue) SYT1 variants. Graph indicates allele frequency of population missense variants in SYT1 from GNOMAD v4.10 (note logarithmic scale, average population allele read number is 1,611,866). No population missense variants are present at putative pathogenic residues. (**b**) Representative images of immunolabelled neurons transfected with SYT1 variants (tagged with pHluorin). Note that WT refers to cells transfected with wild-type SYT1-pHluorin. Left panels show SYT1-pHluorin-transfected neurons in green and right panels display SYT1 immunofluorescence, with warmer colours indicating more intense staining. Arrowheads indicate transfected (closed) and non-transfected (open) nerve terminals. Scale bar = 5 μm. (**c**) SYT1 expression levels at nerve terminals, expressed as the SYT1 immunofluorescence intensity in transfected neurons, relative to non-transfected neurons in the same field of view. Data displayed as mean ± SEM, n = 8–12 (and see Supplementary Table S2a). Kruskal–Wallis test with Dunn's multiple comparison test compared to WT (n = 12); L159R p = 0.0076 (indicated by ∗∗; n = 8), T196K p > 0.99 (12), E209K p > 0.99 (11), E219Q p > 0.99 (11), M303V p > 0.99 (11), S309P p > 0.99 (10), Y365C p > 0.99 (12), G369D p > 0.99 (12), I368T p = 0.60 (12). For all experiments, ‘n’ refers to an individual field of view from an independent coverslip. All experiments were repeated across at least 3 independent cultures, with each culture comprising at least 3 embryos.

We first explored whether newly-identified SYT1 variants (Fig. 1a) impact the expression of the protein in neurons (Fig. 1b). When transfected into neuronal cultures, the majority of SYT1 variants were expressed at a similar level to the control WT protein (Fig. 1c), which suggests that protein stability is not compromised by most variants. Additionally, SYT1 levels in transfected cells (containing both exogenous protein and endogenous WT protein) were approximately twice as high as that observed in untransfected cells (with endogenous WT protein only) for most variants. This equates to an approximately equal copy number of exogenously introduced variant and endogenous WT protein, thereby mimicking the heterozygous expression of these variants. However, the L159R variant is a notable exception, as synaptic levels of this variant were significantly lower than that observed with the WT protein (Fig. 1c). Somatic SYT1 levels also trended towards being decreased in L159R-transfected cells, though were not significantly different to WT (Supplementary Fig. S1). The L159R variant introduces a charged, polar arginine that faces into the hydrophobic interior of the C2A domain, which molecular dynamics simulations predicted may cause notable, diffuse structural perturbations within the domain.^30^ Together, these lines of evidence indicate that L159R likely disrupts SYT1 stability.

**Fig. 2:**
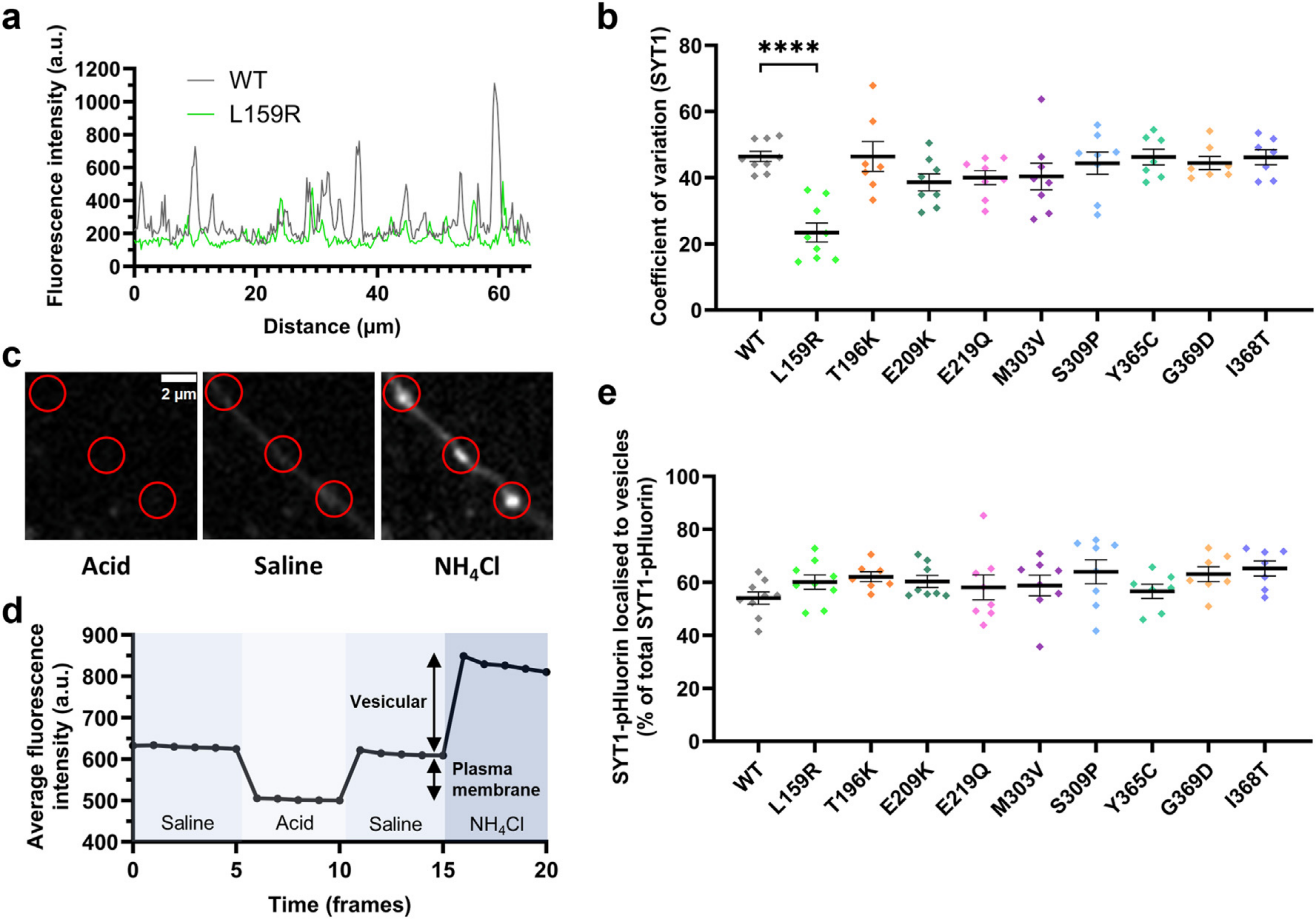
**Most SYT1 variants are trafficked to and distributed within the presynaptic nerve terminal as efficiently as WT protein.** Hippocampal neurons transfected with SYT1-pHluorin variants or wild-type SYT1-pHluorin were sequentially perfused with saline, acidic, or ammonia buffers. (**a**) Representative SYT1-pHluorin fluorescence traces for CV analysis. Peaks in fluorescence intensity indicate higher densities of SYT1. High CV equates to a punctate localisation of SYT1, indicative of enrichment at presynaptic nerve terminals. (**b**) Localisation of SYT1 variants to nerve terminals expressed as CV. Data displayed as mean ± SEM, n = 7–9 (individual field of view from an independent coverslip, from at least 3 independent cultures each comprising at least 3 embryos; and see Supplementary Table S2b). One-way ANOVA with Dunnett's multiple comparison test compared to WT (n = 9); L159R p = <0.0001 (indicated by ∗∗∗∗; n = 9), T196K p > 0.99 (7), E209K p = 0.27 (8), E219Q p = 0.49 (8), M303V p = 0.56 (8), S309P p > 0.99 (8), Y365C p > 0.99 (7), G369D p > 0.99 (7), I368T p > 0.99 (7). (**c**) Representative images of membrane partitioning assay using SYT1-pHluorin. Perfusion with acidic MES buffer quenches all pHluorin fluorescence, while perfusion with saline and ammonia buffers reveal the fluorescence of plasma membrane-bound and total synaptic SYT1-pHluorin, respectively. Circled in red are examples of synaptic ROIs chosen for analysis. Scale bar = 2 μm. (**d**) Protocol and representative data for membrane partitioning assay to distinguish between plasma membrane-bound and synaptic vesicle-localised SYT1. (**e**) Vesicular localisation of SYT1 variants within the presynaptic nerve terminal compared to WT, expressed as a percentage of total SYT1-pHluorin fluorescence in the nerve terminal. Data displayed as mean ± SEM, n = 7–9 (individual field of view from an independent coverslip, from at least 3 independent cultures each comprising at least 3 embryos; and see Supplementary Table S2c). Kruskal–Wallis test with Dunn's multiple comparison test compared to WT (n = 9); L159R p > 0.99 (n = 9), T196K p = 0.42 (7), E209K p > 0.99 (8), E219Q p > 0.99 (8), M303V p > 0.99 (8), S309P p = 0.15 (8), Y365C p > 0.99 (7), G369D p = 0.23 (7), I368T p = 0.09 (7).

We next used coefficient of variation (CV) analysis to investigate whether transport of the protein to nerve terminals is impacted by these variants. Measuring the variation in fluorescence intensity along neurites provides an indication of the distribution of exogenously introduced SYT1 variants along axons and synapses. Most SYT1 variants displayed a punctate distribution, indicative of efficient presynaptic targeting of the protein, and had a similar CV to the WT protein (Fig. 2b). However, the L159R variant was more diffusely localised, displaying a significantly lower CV than WT (Fig. 2a,b), which is in keeping with its lower nerve terminal expression (Fig. 1c). We then ascertained whether SYT1 variants are correctly trafficked to synaptic vesicles, their functional site of action. SYT1 variants were tagged at their lumenal domain with pHluorin, a pH-sensitive GFP which has quenched fluorescence in acidic environments, such as the lumen of synaptic vesicles. We sequentially perfused neurons with saline, acidic, or ammonia buffer to differentiate the partitioning of SYT1 variants to distinct membrane compartments (Fig. 2c,d). All SYT1 variants displayed a similar targeting to vesicles as the WT protein (Fig. 2e). Combined, these results suggest that the majority of C2A and C2B variants do not affect the expression or localisation of SYT1 protein within neurons; however, the L159R variant substantially reduces synaptic levels of SYT1.

### Effect of SYT1 variants on evoked exocytosis

**Fig. 3:**
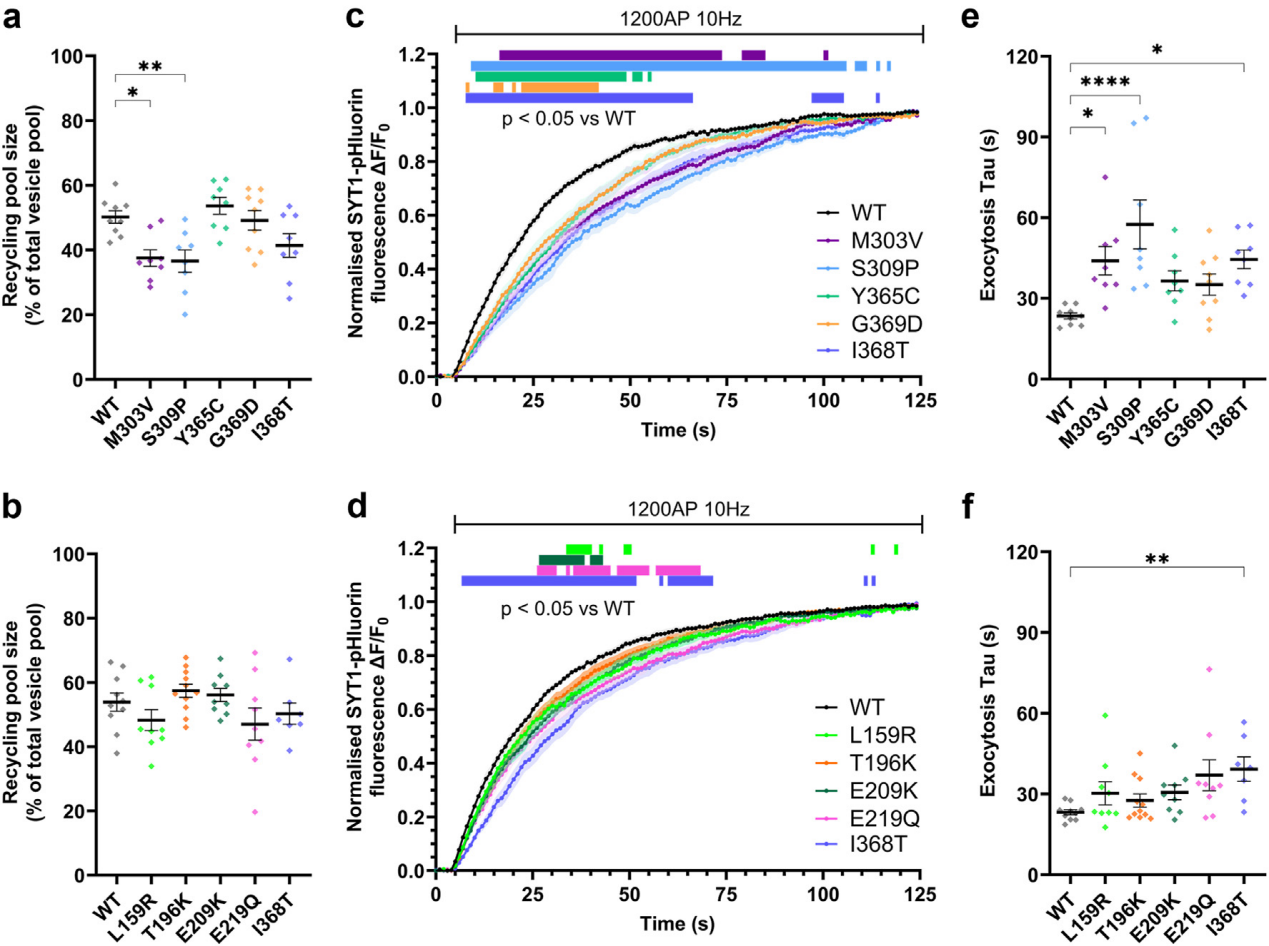
**C2B and C2A domain SYT1 variants induce a dominant-negative slowing of exocytosis.** Hippocampal neurons transfected with SYT1-pHluorin variants or wild-type SYT1-pHluorin were stimulated with 1200 AP at 10 Hz in the presence of bafilomycin A1, and then perfused with ammonia buffer to reveal total SYT1-pHluorin fluorescence. (**a, b**) Synaptic vesicle recycling pool sizes in the presence of C2B (a) or C2A (b) domain variants, expressed as a percentage of the total pool of vesicles (peak SYT1-pHluorin fluorescence with ammonia buffer). One-way ANOVA with Dunnett's multiple comparison test compared to WT; a: M303V p = 0.014, S309P p = 0.0077, Y365C p = 0.87, G369D p = 0.99, I368T p = 0.14. b: L159R p = 0.60, T196K p = 0.87, E209K p = 0.98, E219Q p = 0.41, I368T p = 0.91. (**c, d**) Time course of mean ± SEM ΔF/F_0_ of C2B (c) or C2A (d) domain SYT1-pHluorin variants normalised to peak amplitude of fluorescence change induced by stimulation. SEM indicated by shading. Coloured bars above graph represent time points of significant difference between the SYT1 variants and WT, analysed through repeated measures ANOVA with Dunnett's multiple comparison test. (**e, f**) One-phase curves were fit over the entire stimulation period, from which tau constant values were obtained; tau values shown for C2B (e) or C2A (f) domain variants. e) One-way ANOVA with Dunnett's multiple comparison test compared to WT for C2B variants; M303V p = 0.021, S309P p = <0.0001, Y365C p = 0.23, G369D p = 0.30, I368T p = 0.017. f) Kruskal–Wallis test with Dunn's multiple comparison test compared to WT for C2A variants; L159R p = 0.84, T196K p > 0.99, E209K p = 0.23, E219Q p = 0.074, I368T p = 0.0092. All data displayed as mean ± SEM, n = 7–11 (individual field of view from an independent coverslip, from at least 3 independent cultures each comprising at least 3 embryos; WT n = 9 for a, c, e, n = 10 for b, d, f; I368T n = 8 for a, c, e, n = 7 for b, d, f; M303V n = 8; S309P = 8; Y365C = 8; G369D = 9; L159R = 9; T196K = 11; E209K = 9; E219Q = 9). In a, b, e, f ∗ indicates p < 0.05, ∗∗ indicates p < 0.01, ∗∗∗∗ indicates p < 0.0001 compared to WT; also see Supplementary Table S2d–g.

Having established that most variants are unlikely to affect the levels of SYT1 at nerve terminals, we next explored whether these variants had dominant-negative impacts on SYT1 function. To do this, we used our heterozygous model system, where neurons harbour both exogenously introduced SYT1 variants and endogenous WT SYT1. Firstly, we investigated whether any variants impact the total availability of synaptic vesicles for exocytosis. Neurons were stimulated with 1200 AP at 10 Hz in the presence of bafilomycin A1 to block vesicle reacidification. Increases in pHluorin fluorescence thus provides a measure of cumulative vesicle exocytosis. This approach was used to establish the proportion of vesicles that are mobilised by the stimulus train, which comprises the recycling pool of vesicles (Supplementary Fig. S2). The presence of SYT1 C2B domain variants M303V and S309P significantly reduced the size of the recycling pool compared to WT (Fig. 3a) whereas all C2A variants (Fig. 3b) and remaining C2B variants (Fig. 3a) did not impact the total availability of synaptic vesicles for exocytosis.

We then explored whether the SYT1 variants had any dominant-negative impacts on the kinetics of neurotransmitter release. The cumulative mobilisation of recycling pool vesicles was ascertained from pHluorin fluorescence time traces, normalised to the maximal fluorescence induced by stimulation (Fig. 3c,d). The presence of either SYT1 C2B (Fig. 3c) or C2A (Fig. 3d) domain variants impaired evoked exocytosis of recycling pool vesicles in a dominant-negative manner. Notably, SYT1 variants produced graded impairments to neurotransmitter release, as evidenced by the number of time points where they are significantly different to WT (see bars denoting significance in Fig. 3c,d, though note that T196K is not significantly different to WT). In particular, the C2B domain variants generally had a more pronounced effect than the C2A variants (Fig. 3c,d). This was further demonstrated through analysis of time constant (tau) values extracted from one-phase curves fit to the time courses as a measure of overall exocytic rate. Among the C2B domain variants, M303V and S309P caused a significant decrease in the overall rate of mobilisation of the recycling pool of vesicles, as was also observed for the previously-characterised I368T variant (Fig. 3e). None of the C2A domain variants had exocytic taus that significantly differed from WT (Fig. 3f), though all trended towards being increased.

**Fig. 4:**
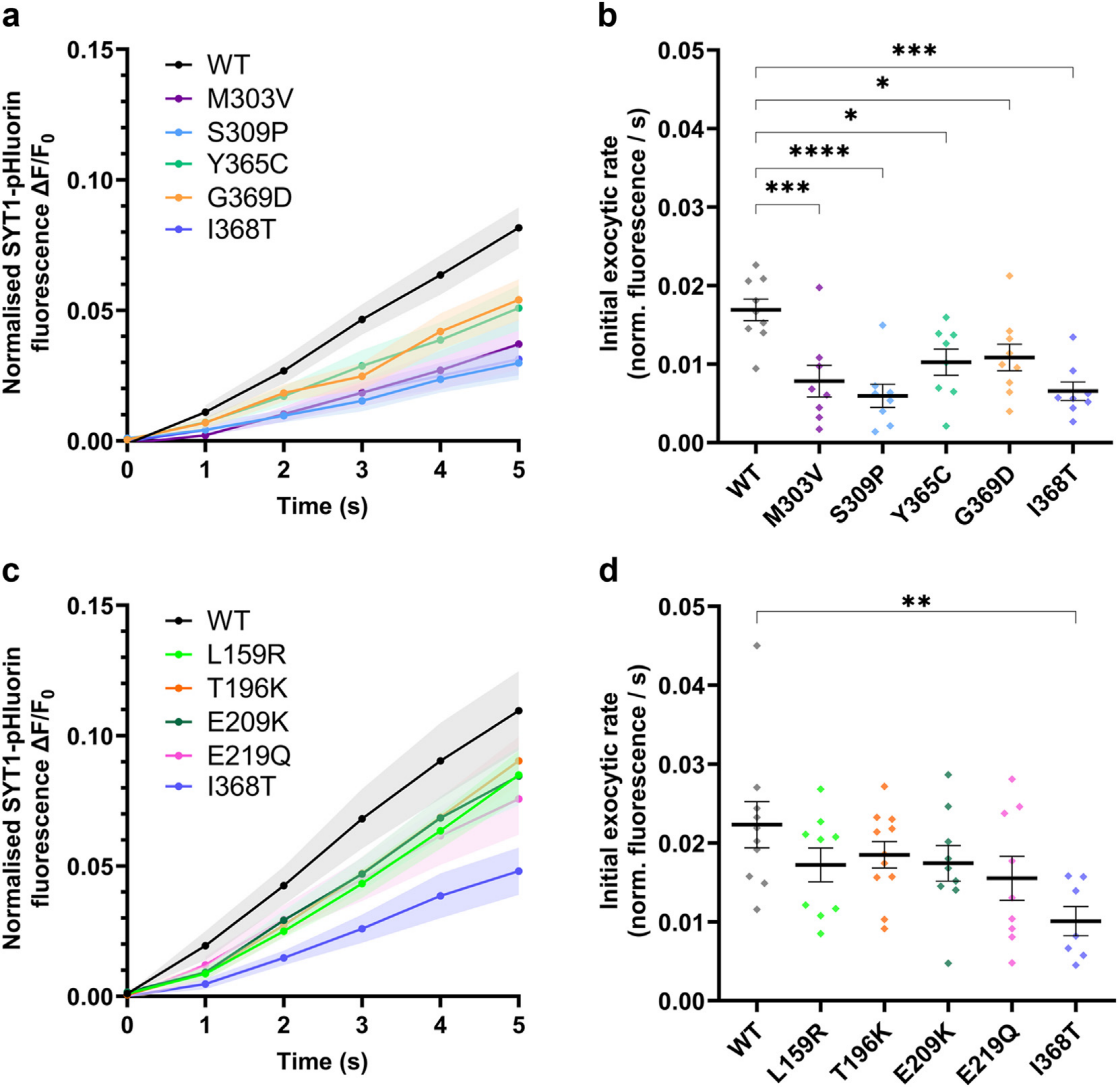
**Impact of SYT1 variants on initial exocytic rate.** Hippocampal neurons transfected with SYT1-pHluorin variants or wild-type SYT1-pHluorin were stimulated with 1200 AP at 10 Hz in the presence of bafilomycin A1 and perfused with ammonia buffer to reveal total SYT1-pHluorin fluorescence. (**a, c**) Time course (first 5 s of stimulation) of mean ΔF/F_0_ of C2B (a) or C2A (c) domain SYT1-pHluorin variants normalised to maximum fluorescence induced by ammonia buffer. SEM indicated by shading. (**b, d**) Initial exocytic rates for C2B (b) or C2A (d) domain variants over the first 5 s of stimulation. One-way ANOVA with Dunnett's multiple comparison test compared to WT; b: M303V p = 0.0009, S309P p = <0.0001, Y365C p = 0.020, G369D p = 0.032, I368T p = 0.0001. d: L159R p = 0.40, T196K p = 0.63, E209K p = 0.44, E219Q p = 0.16, I368T p = 0.0048. Data displayed as mean ± SEM, n = 7–11 (individual field of view from an independent coverslip, from at least 3 independent cultures each comprising at least 3 embryos; WT n = 9 for a, b, n = 10 for c, d; I368T n = 8 for a, b, n = 7 for c, d; M303V n = 8; S309P = 8; Y365C = 8; G369D = 9; L159R = 9; T196K = 11; E209K = 9; E219Q = 9). In b, d ∗ indicates p < 0.05, ∗∗p < 0.01, ∗∗∗p < 0.001, and ∗∗∗∗p < 0.0001 compared to WT; also see Supplementary Table S2h and i

To gain a better appreciation of how SYT1 variants exert their pathogenic effects, we specifically examined synaptic vesicle exocytosis over the initial 5 s of stimulation when the exocytic rate is largely linear. For these analyses, we examined the rate of fusion relative to the entire pool of vesicles, to account for any changes in recycling pool size (Supplementary Fig. S2). All C2B domain SYT1 variants demonstrated a significant slowing of initial exocytic rate compared to the WT protein (Fig. 4a,b). Graded effects on exocytosis kinetics were again observed across these variants; M303V and S309P variants decreased the initial rate of exocytosis to a similar extent as the I368T reference variant, whereas Y365C and G369D exhibited a less severe effect (Fig. 4a,b). The presence of C2A domain variants induced a modest reduction in initial exocytic rate, but this effect was not statistically significant (Fig. 4c,d). Together with the findings from Fig. 3c,d, these findings suggest that SYT1 variants in both the C2A and C2B domains impair synaptic vesicle exocytosis; however, they do so to varying degrees, with C2B domain variants having substantially greater impacts on evoked exocytosis compared to C2A domain variants.

**Fig. 5:**
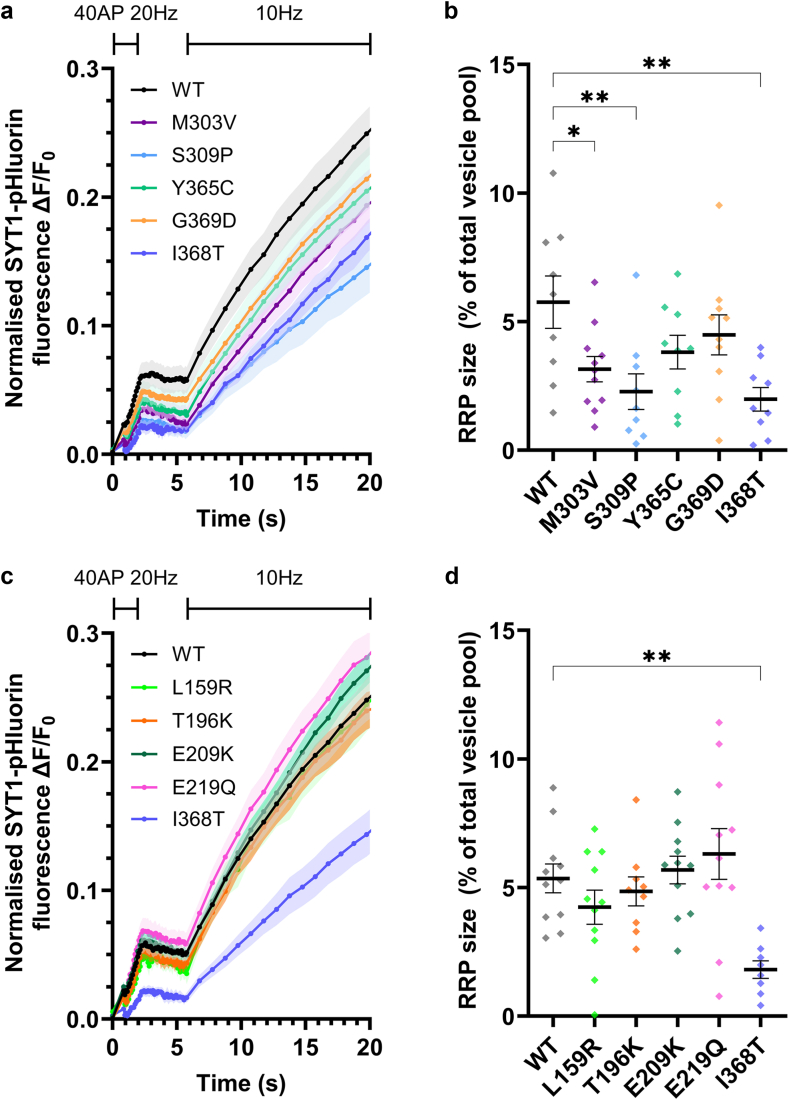
**Impact of SYT1 variants on the readily releasable pool (RRP) of vesicles.** Hippocampal neurons transfected with SYT1-pHluorin variants or wild-type SYT1-pHluorin were stimulated with 40 AP at 20 Hz in the presence of bafilomycin A1, rested for 3 s, then stimulated continuously at 10 Hz (to identify active synapses) before being perfused with ammonia buffer to reveal total SYT1-pHluorin fluorescence. (**a, c**) Time course of mean ΔF/F_0_ of C2B (a) or C2A (c) domain SYT1-pHluorin variants normalised to maximum fluorescence induced by ammonia buffer. SEM indicated by shading. (**b, d**) Size of RRP (% of vesicles fused by 40AP) in the presence of C2B (b) or C2A (d) variants as a proportion of total vesicles revealed by ammonia buffer. One-way ANOVA with Dunnett's multiple comparison test compared to WT; b: M303V p = 0.040, S309P p = 0.0053, Y365C p = 0.21, G369D p = 0.57, I368T p = 0.0022. d: L159R p = 0.63, T196K p = 0.98, E209K p = 0.99, E219Q p = 0.75, I368T p = 0.0034. Data displayed as mean ± SEM, n = 8–11 (individual field of view from an independent coverslip, from at least 3 independent cultures each comprising at least 3 embryos; WT n = 9 for a, b, n = 11 for c, d; I368T n = 9 for a, b, n = 8 for c, d; M303V n = 11; S309P = 9; Y365C = 9; G369D = 10; L159R = 11; T196K = 9; E209K = 11; E219Q = 11). In b, d ∗ indicates p < 0.05 and ∗∗p < 0.01 compared to WT; also see Supplementary Table S2j and k.

To gain mechanistic insight into how SYT1 variants may be driving their impacts, we next measured the size of the readily releasable pool (RRP) of vesicles (i.e. those vesicles docked and primed at the plasma membrane) in the presence of SYT1 variants. To do this, neurons transfected with WT or variant SYT1 were stimulated with a short, high frequency burst of action potentials (40 AP, 20 Hz) which triggers exocytosis of RRP vesicles,^47^, ^48^, ^49^ and then further stimulated at 10 Hz to identify active boutons (Fig. 5a,c). SYT1 C2B variants were observed to have a graded impact on the RRP; the RRP was significantly reduced in the presence of M303V, S309P and I368T SYT1, and there was a trend towards a smaller RRP in the presence of Y365C SYT1 (Fig. 5a,b). In contrast, the RRP was unaffected in the presence of SYT1 C2A variants (Fig. 5c,d).

### Associations between presynaptic functional deficits and phenotyping measures for participants with SYT1 variants

**Fig. 6:**
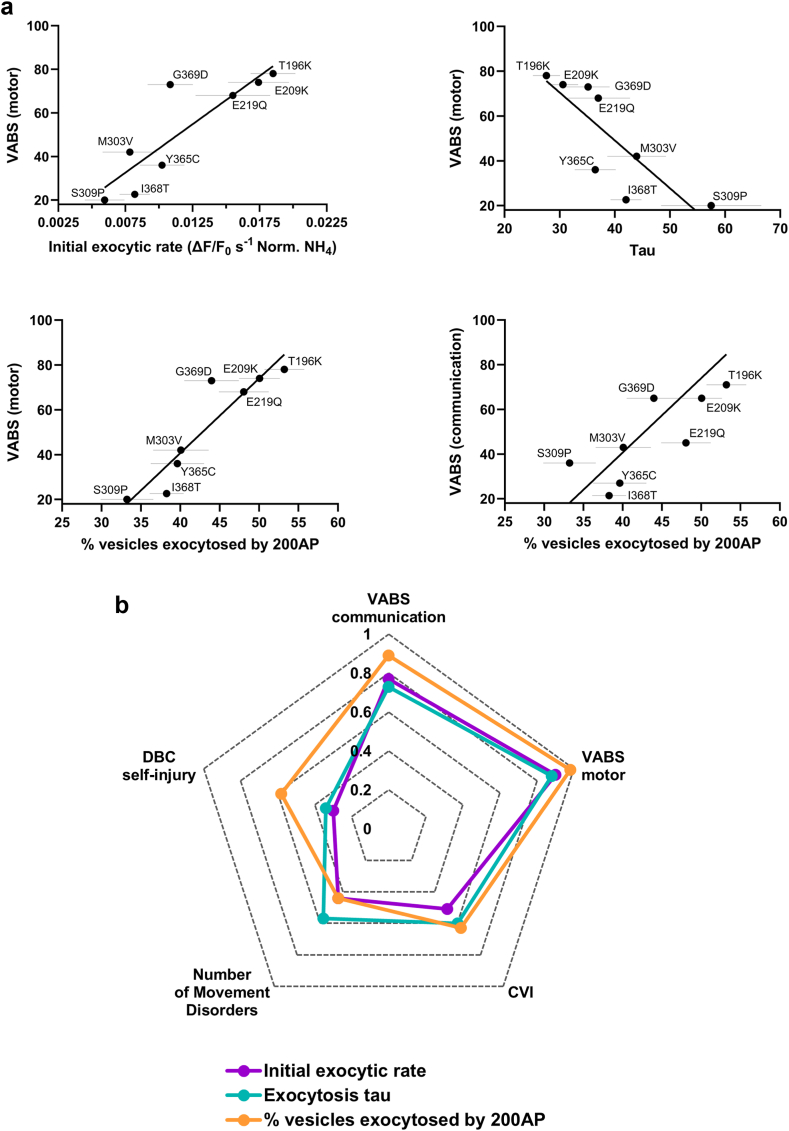
**Exocytic efficiency in the presence of SYT1 variants strongly correlates with motor and communication phenotypes of SYT1-associated neurodevelopmental disorder.** Quantitative phenotypes exhibited by individuals harbouring SYT1 variants were each assessed for correlation with 3 separate functional measures of exocytosis kinetics—initial exocytic rate (over first 5 s of 10 Hz stimulation), overall exocytic rate (tau), and percentage of vesicles fused by 200 AP (Supplementary Fig. S3). Phenotyping measures include motor and communication subscales of the Vineland Adaptive Behaviour Scale (VABS), number of movement disorders, self-injury scores from the Developmental Behaviour Checklist (DBC), and cerebral visual impairment (CVI). (**a**) Scatter plots of significant correlations between exocytic measures in the presence of SYT1 variants and quantitative neurodevelopmental phenotypic measures of individuals with SYT1 variants. Gray horizontal lines indicate SEM with regards to exocytic measures. (**b**) Radar plot of correlations between exocytic measures in the presence of SYT1 variants and neurodevelopmental phenotypic measures of individuals with SYT1 variants. Spearman's r coefficients are presented with directionality removed for ease of interpretation.

We next tested whether the relative exocytic efficiency in the presence of SYT1 variants is associated with variation in the neurodevelopmental phenotypes of individuals harbouring these variants. Analyses were focused on neurodevelopmental characteristics which we have previously reported to differ between SYT1 participants and comparison participants with other monogenic intellectual disabilities^30^: communication and motor impairments through subscales of the VABS, CVI, movement disorders, and self-injurious behaviour from the DBC. We explored correlations between quantitative phenotyping data for each of the SYT1 variants examined in this paper (except L159R due to data unavailability) and three different measures of presynaptic function that reflect different aspects of vesicle fusion and neurotransmitter release: initial exocytic rate within the first 5 s, overall exocytic rate (tau, equating to rate of mobilisation of the recycling pool), and percentage of vesicles fused by 200 AP (timepoint where SYT1 variants induced their strongest impacts, Supplementary Fig. S3). Table 1 shows permutation correlation test results for associations between phenotypic measures and metrics of synaptic vesicle exocytosis. Strong correlations were found between the percentage of vesicles exocytosed by 200 AP and age-standardised scores on both the Vineland motor and the Vineland communication sub-scales (Table 1, Fig. 6a,b), such that lower evoked exocytosis correlated with more severe impairments in adaptive functioning. Strong correlations were also found between the Vineland motor sub-scale and both the initial exocytic rate and exocytic tau, where a lower initial exocytic rate or a slower tau, respectively, correlated with more severe motor deficits (Table 1, Fig. 6a,b). In contrast, no metric of presynaptic function significantly correlated with the number of movement disorder types reported, DBC self-injury sub-scale, or CVI score (Table 1, Fig. 6b, Supplementary Fig. S6–S8).

**Table 1:** Spearman's correlations between exocytosis metrics and participant phenotyping measures.

	Initial exocytic rate (norm. (ΔF/F_0_)/s)				Exocytosis tau (s)				% vesicles fused by 200AP			
	r	p	95% CI		r	p	95% CI		r	p	95% CI	
VABS communication	0.77	0.028	0.1	1	−0.73	0.042	−1	0.06	0.89	0.004∗	0.39	1
VABS motor	0.90	0.002∗	0.41	1	−0.88	0.005∗	−1	0.06	0.98	<0.001∗	0.73	1
CVI	−0.51	0.24	−1	0.47	0.60	0.16	−0.34	1	−0.63	0.13	−1	0.29
Number of Movement Disorders	−0.44	0.25	−0.94	0.46	0.57	0.14	−0.45	0.94	−0.44	0.25	−0.94	0.52
DBC Self-Injury	0.25	0.59	−0.87	0.96	0.04	0.94	−0.88	0.96	0.07	0.88	−0.87	0.96

Asterisks indicate significant results after corrections.

Significance threshold at p ≤ 0.019 to account for multiple comparisons.

VABS, Vineland Adaptive Behaviour Scale; DBC, Developmental Behaviour Checklist; CVI, cerebral visual impairment.

## Discussion

**Fig. 7:**
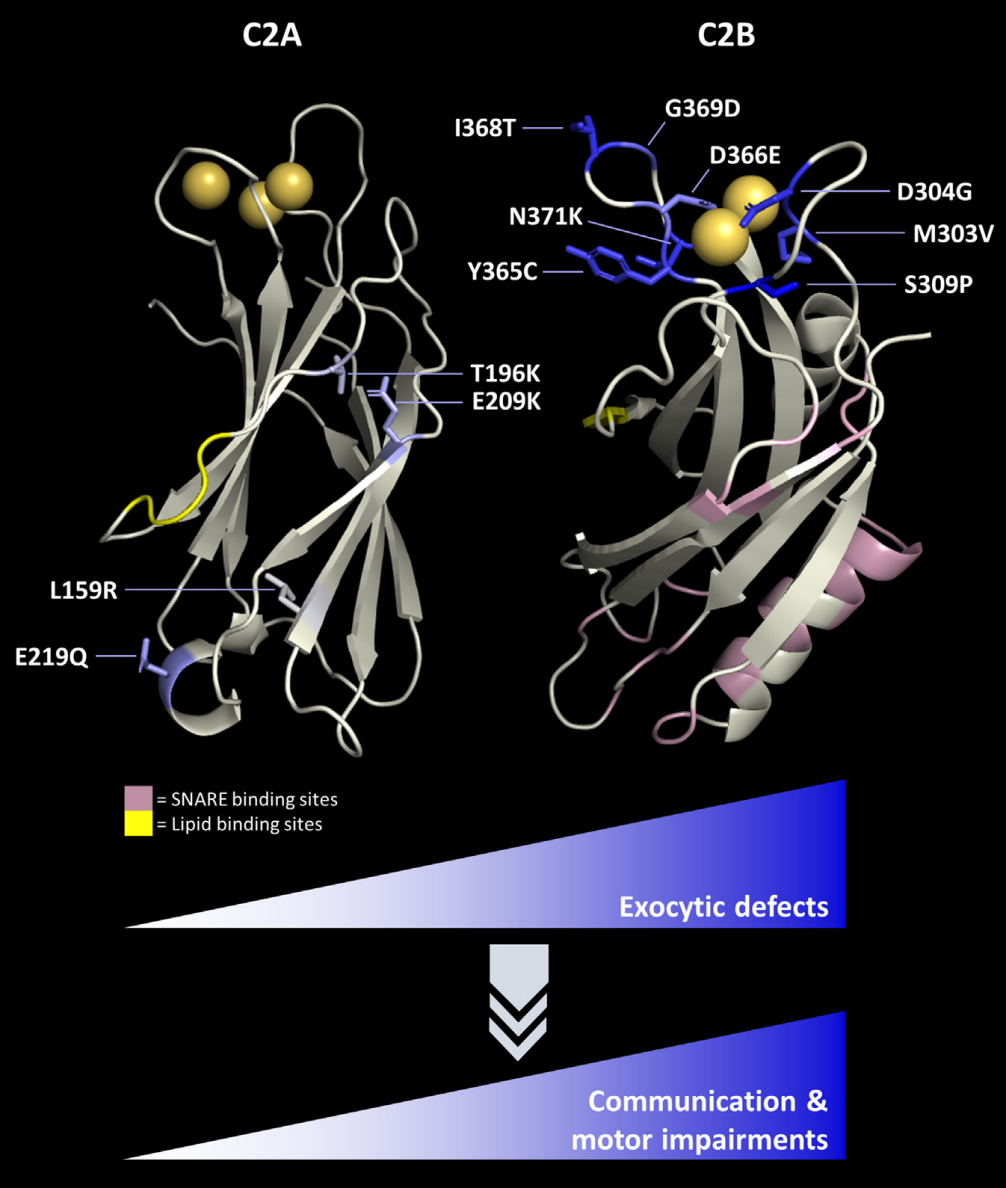
**Genotype-function-phenotype relationship in SYT1-associated neurodevelopmental disorder**. SYT1 variants in both the C2A domain (top left) and C2B domain (top right) cause graded, dominant-negative impairments to evoked exocytosis (this paper,^29^ colour of each residue equates to relative impact to exocytosis, gold spheres are calcium ions). The degree of impaired exocytosis correlates with motor and communication difficulties in individuals harbouring these variants, and is dependent on the nature of the amino acid substitution and its location within SYT1. SYT1 variants that perturb protein stability tend to present with stronger adaptive functions and fewer neurological symptoms, which may be due to either reduced expression or mitigation of dominant-negative effects. Known regions of functional importance are also indicated: polybasic lysine patches which bind to acidic phospholipids (yellow),^20^ and residues of the primary and tripartite interfaces between SYT1 and its SNARE protein binding partners (pink).^23^^,^^50^

Here, we have established the pathogenicity of newly identified SYT1 C2B and C2A domain variants. We show that variants in disparate regions of SYT1 share a common underlying mechanism of pathogenicity, causing a graded dominant-negative impairment in synaptic vesicle exocytosis. Importantly, we have also explored genotype-function-phenotype relationships for these variants, demonstrating that lower exocytic efficiency correlates with motor and communication difficulties in individuals harbouring these variants (Fig. 7). This establishes impairment of evoked neurotransmitter release as a likely candidate mechanism constraining the development of cognitive and motor abilities, leading to core features of SYT1-associated neurodevelopmental disorder.

We demonstrate that four recently identified SYT1 C2B domain variants impair synaptic vesicle exocytosis in a dominant-negative manner, a pathogenic mechanism previously established for other SYT1 C2B variants.^28^^,^^29^ Evoked exocytosis was reduced over both shorter and prolonged periods of stimulation in the presence of the C2B variants, though the degree of impact was variable. The position of variants within the Ca^2+^-binding pocket of the C2B domain may contribute to this variability. Variants located in Ca^2+^-binding loop 1 (M303V, S309P (this study) and D304G^29^) cause severe exocytic defects, whereas some loop 3 variants, including Y365C, G369D (this study) and D366E,^29^ induced milder exocytic impairments. However, this is not axiomatic: N371K and I368T (this study and ^29^), which induce comparatively severe exocytic deficits, also reside in loop 3. Moreover, alternative variants at a recurrent locus can have differential effects. M303K was previously shown to impair the stability and expression of SYT1,^29^ an effect not recapitulated by M303V in this current study. Intriguingly, these variants are also associated with different neurodevelopmental characteristics.^29^^,^^30^ Therefore, the functional severity, or indeed mechanism of action, of variants cannot be simply predicted by residue location alone.

Importantly, we provide functional evidence for the pathogenicity of C2A domain variants identified in individuals with neurodevelopmental delay. C2A domain variants act in a similar fashion to C2B variants, impairing synaptic vesicle exocytosis in a dominant-negative manner. However, C2A domain variants had comparatively modest impacts and did not significantly reduce the initial exocytic rate; instead, significant divergence from WT SYT1 only emerged with extended stimulation (Fig. 3d). Overall, the severity of exocytic impairment induced by all four C2A variants was milder than the comparison C2B I368T variant. It is noteworthy, however, that E219Q had the greatest impact among the C2A variants, as evidenced by the time course of cumulative exocytosis where E219Q SYT1 demonstrated the longest period of significant difference compared to WT (Fig. 3d). Correspondingly, the individual harbouring this variant also presented with more severe impairments in adaptive behaviour than the other C2A variants.^30^ The severity of this particular C2A variant was not predictable from molecular modelling, highlighting the power of functional interrogation of SYT1 variants to not only establish their mechanism of pathogenicity, but also for predicting neurodevelopmental phenotypes in SYT1-associated neurodevelopmental disorder.

These findings suggest that the impact of SYT1 variants in either C2 domain converge on a similar synaptic phenotype, with C2B domain variants having, on average, a greater magnitude of dominant-negative effect on exocytosis compared to C2A domain variants. This observation is in line with the differential proposed roles of the two domains, whereby the C2B domain is essential for triggering evoked exocytosis, while the C2A domain plays an auxiliary role.^16^^,^^17^^,^^19^ However, there is a notable confound to the simple comparison between variants in C2A and C2B domains: in contrast to the C2B variants explored here, all C2A variants in this study are located outside the Ca^2+^-binding loops (Fig. 7). In fact, all investigated C2A variants occur in regions of currently unknown significance to SYT1 function. For most C2A variants (except L159R, as detailed below), molecular dynamics simulations and modelling did not indicate any major impacts on domain structure or function or obvious mechanism of pathogenicity.^30^ Hence, it is perhaps unsurprising that these variants have milder impacts on exocytosis kinetics overall compared to their C2B domain counterparts, most of which involve substitutions to residues of the critical Ca^2+^-binding loops. It remains to be seen whether variants affecting Ca^2+^-binding residues of the C2A domain would result in mild or more severe exocytic defects (and corresponding clinical impacts).

While SYT1 C2A and C2B variants converge to impair exocytosis, they may do so via distinct means. SYT1 C2B domain variants have mutation-specific, graded impacts on the size of the RRP (Fig. 5), suggesting these variants differentially impact either the docking and priming of vesicles or vesicle release probability. These effects may then underlie the impaired exocytosis in the presence of C2B variants. C2A domain variants, however, did not impact RRP size, suggesting that they impair exocytosis via alternative means.

We also explored alternative mechanisms of pathogenicity beyond dominant-negative effects to exocytosis, by examining how SYT1 variants impact protein expression and transport. In keeping with observations from C2B variants studied to date,^28^^,^^29^^,^^34^^[preprint]^ most of the investigated SYT1 variants across the C2A and C2B domains had no impact on nerve terminal expression or trafficking of SYT1, indicating that disruption to protein stability and expression are not common consequences of SYT1 missense variants.

There are, however, exceptions to this rule–the C2A domain variant L159R impaired SYT1 targeting to nerve terminals. L159 lies in a beta-strand and its sidechain faces the hydrophobic interior of the C2A beta-sandwich structure. Substituting a hydrophobic sidechain (leucine) for a positively charged sidechain (arginine) was predicted via molecular dynamic simulations to introduce structural instability to multiple regions of the C2A domain.^30^ The aforementioned M303K C2B domain variant, which also increased structural instability, was similarly expressed at lower levels.^29^ Interestingly, from qualitative reports^29^^,^^30^^,^^51^ both variants are associated with a relatively mild neurodevelopmental phenotype. It is therefore possible that these two variants confer haploinsufficiency and/or have a limited ability to exert deleterious dominant negative effects in neurons. The latter argument is supported by the current evidence that, despite decreased synaptic levels, L159R expression in cell soma was not significantly lower than WT (though did trend towards being decreased, Supplementary Fig. S1), and some copies of SYT1 L159R can traffic to vesicles and can exert dominant-negative slowing of exocytosis, albeit mild. Thus, amino acid substitutions to diverse regions of the protein can have major impacts to stability, expression and/or trafficking, likely dependent on the nature of the variant. Together, these findings indicate that SYT1 variants may have heterogeneous molecular and cellular effects which are not easily predicted by variant location, though dominant-negative impairment of exocytosis remains the most frequent pathogenic mechanism.

This study has specifically investigated missense variants that were selected because they 1) fall within different domains of the protein, 2) reside in different regions of each domain, and/or 3) are predicted to have diverse impacts on protein structure. Further insights will be gained by investigating a greater number of variants, increasing power for correlational analyses. For example, it would be important to explore whether a recently identified C2A variant (V184A) in an individual with a severe, early-lethal neurodevelopmental disorder,^32^ causes severe impairment to evoked neurotransmitter release. Moreover, we have only assessed the impacts of these variants on evoked exocytosis; previously investigated SYT1 C2B variants slow evoked exocytosis without having dominant-negative impacts on the additional functions of SYT1 as a clamp for spontaneous neurotransmitter release and a modulator of endocytosis.^34^^[preprint]^ It therefore remains possible that the newly-identified SYT1 variants may additionally impact these other presynaptic processes.

We have shown that impacts on evoked exocytosis are likely the primary mechanism via which SYT1 variants confer their pathogenicity. How could these SYT1 variants impair neurotransmitter release from a molecular perspective? SYT1 has multiple binding partners including the SNARE complex proteins, complexin, and plasma membrane phospholipids, and efficient interaction with these partners is crucial for vesicle exocytosis.^15^^,^^52^^,^^53^ Strategic variants of SYT1 that interfere with these interactions result in impaired exocytosis.^9^^,^^23^^,^^54^, ^55^, ^56^ A selection of pathogenic SYT1 variants (D366E, D304G, I368T) have been shown to reduce Ca^2+^-dependent membrane binding.^31^ Interestingly, the degree of binding defect induced by these variants corresponded with the severity of their impact to neurotransmission.^31^ Further studies should therefore ascertain whether the pathogenic SYT1 variants investigated here also impair interactions with the native SYT1 binding partners, which may underlie the exocytic effects observed in this study.

Nevertheless, the strong correlations observed between exocytic efficiency and severity of adaptive impairments support disruption to evoked neurotransmitter release as a primary pathogenic mechanism underlying SYT1-associated neurodevelopmental disorder. We have previously observed that three individuals harbouring the SYT1 C2B variant D366E presented with milder motor delay than those harbouring other SYT1 variants, and did not exhibit the early-onset movement disorders seen in other members of the cohort; this variant also induced the mildest impact on evoked exocytosis among those variants examined.^29^ However, these observations were based on non-standardised, qualitative reporting of clinical phenotypes and only a single variant associated with milder exocytic defects and clinical presentations. In the present study, we aggregated presynaptic function and quantitative phenotyping data for 8 SYT1 variants across the two C2 domains, which demonstrated a significant correlation between standardised scores of motor and communication skills and metrics of exocytic efficacy. This represents robust evidence for a unifying functional impairment that underlies and drives these adaptive impairments.

We have focused our study on a limited range of phenotypes that we have previously detailed to be prevalent in SYT1-associated neurodevelopmental disorder, and for which quantitative measures were available. The observed function-phenotype correlations did not extend to all phenotype measures, with no significant correlations between impairments in exocytosis observed *in vitro* and the number of movement disorders, CVI scores or self-harm items of the DBC. Methodological limitations include the lack of sensitivity of carer-report measures for these phenotypic dimensions, and limited power to detect significant associations in a small group size, especially for complex behavioural characteristics which will be subject to modifying influences and changes over time. Practical limitations imposed by geographic spread of study participants meant that all data were acquired online via standardised research protocol, rather than in-person assessment. We also note that for most variants phenotyping data were only available for a single individual, though for recurrent variants phenotypic variation has been shown to be remarkably consistent.^30^ Thus, the absence of significant correlations should be interpreted with caution and does not preclude mechanistic relationships for these symptom domains. There may be unmeasured confounds of within-group associations, including medication exposures or medical co-morbidities, which require investigation in larger samples.^30^ However, if the observed contrasts in function-phenotype associations are later confirmed, this could reflect diverse mechanisms contributing to different aspects of the condition.

Our results inform knowledge of the mechanism of dysfunction caused by SYT1 variants at the level of single synapses, whilst the complex SYT1-associated neurodevelopmental phenotypes implicate widespread brain networks maturing across development. What then might link impaired exocytosis to motor and communication difficulties? There are several possible explanations. Reduced neurotransmitter release may influence synaptogenesis and/or embryonic formation of local and distributed neuronal circuitry.^57^^,^^58^ However, major disruptions to SYT1 function do not substantially alter neuronal pathfinding, or synapse number and morphology in multiple cellular and *in vivo* mammalian and invertebrate model systems,^4^^,^^11^^,^^26^^,^^59^, ^60^, ^61^, ^62^ though SYT1 has been shown to positively influence axonal branching in an avian model.^63^ Notably, even in the absence of evoked neurotransmitter release, such as in prenatal SNAP-25 knockout mice, axonal growth and thalamocortical development is largely unperturbed.^64^

A more likely possibility is that dampening of evoked synchronous release reduces the fidelity of neurotransmission. Impaired evoked neurotransmitter release would be expected to have an ongoing impact on synaptic strength and plasticity, blunting processes such as long-term potentiation, impairing learning and memory and thereby leading to constraints on the acquisition and optimisation of motor and cognitive skills.^65^ The impacts that this has on discrete circuits and thus behaviours would depend on the relative contribution of SYT1 to these. There are up to 17 members of the synaptotagmin family, and in particular SYT2, SYT7 and SYT9 replace SYT1 as the major sensors for fusion in a number of central neuronal cell types.^66^, ^67^, ^68^, ^69^ Reliance on SYT1, or sensitivity to impaired evoked synchronous release, therefore likely varies between specific cell types, circuits, brain regions and developmental time-windows.^70^, ^71^, ^72^, ^73^ Therefore, impaired SYT1 function is expected to lead to diverse impacts to neural networks and circuits causing varied behavioural impairments that may change across the lifespan.

Further *in vivo* experimental evidence is required to resolve the multi-level mechanisms bridging between parameters of presynaptic function and the emergent properties of motor control and cognitive development. Developing *in vivo* animal models harbouring neurodevelopmental disorder-associated SYT1 variants will be key to understanding how these affect whole-brain, network-based systems. These models could subsequently be used to explore whether elements of SYT1-associated neurodevelopmental disorder can be ameliorated. With the finding that perturbed exocytosis is common to both C2A and C2B variants, and correlates with disorder severity, this emerges as a core tractable target to investigate for therapeutic amelioration of this disorder.

## Contributors

Conceptualisation: S.L.G, K.B. and P.Y.P.; Data Curation: P.Y.P., L.E.B., J.E.; Formal Analysis: P.Y.P., L.E.B., N.S., R.A.J.; Funding Acquisition: S.L.G., K.B., L.E.B.; Investigation: P.Y.P., L.E.B., N.S., M.A.A., J.E., R.A.J.; Visualisation: P.Y.P. and L.E.B.; Writing-original draft: P.Y.P. (lead), S.L.G.; Writing-review and editing: P.Y.P., S.L.G., K.B., L.E.B., H.M. All authors reviewed and approved the final version of the manuscript.

## Data sharing statement

Raw data are available from the point of publication upon request to the corresponding author (sarah.gordon@florey.edu.au). This will be provided upon investigator support (K.B., S.L.G), following approval of a proposal (to K.B., S.L.G) and with a signed data access agreement, and in accordance with ethical guidelines and study participant confidentiality requirements.

## Declaration of interests

The authors declare no competing financial interests.

## References

[1] Melland H., Carr E.M., Gordon S.L. (2021). Disorders of synaptic vesicle fusion machinery. J Neurochem.

[2] Broadie K., Bellen H.J., DiAntonio A., Littleton J.T., Schwarz T.L. (1994). Absence of synaptotagmin disrupts excitation-secretion coupling during synaptic transmission. Proc Natl Acad Sci U S A.

[3] DiAntonio A., Schwarz T.L. (1994). The effect on synaptic physiology of synaptotagmin mutations in Drosophila. Neuron.

[4] Liu H., Dean C., Arthur C.P., Dong M., Chapman E.R. (2009). Autapses and networks of hippocampal neurons exhibit distinct synaptic transmission phenotypes in the absence of synaptotagmin I. J Neurosci.

[5] Fernandez I., Ubach J., Dulubova I., Zhang X., Sudhof T.C., Rizo J. (1998). Three-dimensional structure of an evolutionarily conserved N-terminal domain of syntaxin 1A. Cell.

[6] Fernandez-Chacon R., Konigstorfer A., Gerber S.H. (2001). Synaptotagmin I functions as a calcium regulator of release probability. Nature.

[7] Mackler J.M., Drummond J.A., Loewen C.A., Robinson I.M., Reist N.E. (2002). The C(2)B Ca(2+)-binding motif of synaptotagmin is required for synaptic transmission in vivo. Nature.

[8] Bai J., Wang P., Chapman E.R. (2002). C2A activates a cryptic Ca(2+)-triggered membrane penetration activity within the C2B domain of synaptotagmin I. Proc Natl Acad Sci U S A.

[9] Paddock B.E., Wang Z., Biela L.M. (2011). Membrane penetration by synaptotagmin is required for coupling calcium binding to vesicle fusion in vivo. J Neurosci.

[10] Bai J., Wang C.T., Richards D.A., Jackson M.B., Chapman E.R. (2004). Fusion pore dynamics are regulated by synaptotagmin∗t-SNARE interactions. Neuron.

[11] Geppert M., Goda Y., Hammer R.E. (1994). Synaptotagmin I: a major Ca2+ sensor for transmitter release at a central synapse. Cell.

[12] DiAntonio A., Parfitt K.D., Schwarz T.L. (1993). Synaptic transmission persists in synaptotagmin mutants of Drosophila. Cell.

[13] Littleton J.T., Stern M., Schulze K., Perin M., Bellen H.J. (1993). Mutational analysis of Drosophila synaptotagmin demonstrates its essential role in Ca(2+)-activated neurotransmitter release. Cell.

[14] Nishiki T., Augustine G.J. (2004). Dual roles of the C2B domain of synaptotagmin I in synchronizing Ca2+-dependent neurotransmitter release. J Neurosci.

[15] Wang S., Li Y., Ma C. (2016). Synaptotagmin-1 C2B domain interacts simultaneously with SNAREs and membranes to promote membrane fusion. Elife.

[16] Gruget C., Bello O., Coleman J. (2020). Synaptotagmin-1 membrane binding is driven by the C2B domain and assisted cooperatively by the C2A domain. Sci Rep.

[17] Striegel A.R., Biela L.M., Evans C.S. (2012). Calcium binding by synaptotagmin's C2A domain is an essential element of the electrostatic switch that triggers synchronous synaptic transmission. J Neurosci.

[18] Bowers M.R., Reist N.E. (2020). The C2A domain of synaptotagmin is an essential component of the calcium sensor for synaptic transmission. PLoS One.

[19] Lee J., Guan Z., Akbergenova Y., Littleton J.T. (2013). Genetic analysis of synaptotagmin C2 domain specificity in regulating spontaneous and evoked neurotransmitter release. J Neurosci.

[20] Wu Z., Ma L., Courtney N.A. (2022). Polybasic patches in both C2 domains of synaptotagmin-1 are required for evoked neurotransmitter release. J Neurosci.

[21] Shields M.C., Bowers M.R., Kramer H.L. (2020). The role of the C2A domain of synaptotagmin 1 in asynchronous neurotransmitter release. PLoS One.

[22] Shin O.H., Xu J., Rizo J., Sudhof T.C. (2009). Differential but convergent functions of Ca2+ binding to synaptotagmin-1 C2 domains mediate neurotransmitter release. Proc Natl Acad Sci U S A.

[23] Zhou Q., Zhou P., Wang A.L. (2017). The primed SNARE-complexin-synaptotagmin complex for neuronal exocytosis. Nature.

[24] Wu D., Bacaj T., Morishita W. (2017). Postsynaptic synaptotagmins mediate AMPA receptor exocytosis during LTP. Nature.

[25] Bouazza-Arostegui B., Camacho M., Brockmann M.M., Zobel S., Rosenmund C. (2022). Deconstructing synaptotagmin-1's distinct roles in synaptic vesicle priming and neurotransmitter release. J Neurosci.

[26] Fernandez-Chacon R., Shin O.H., Konigstorfer A. (2002). Structure/function analysis of Ca2+ binding to the C2A domain of synaptotagmin 1. J Neurosci.

[27] Powell C.M., Schoch S., Monteggia L. (2004). The presynaptic active zone protein RIM1alpha is critical for normal learning and memory. Neuron.

[28] Baker K., Gordon S.L., Grozeva D. (2015). Identification of a human synaptotagmin-1 mutation that perturbs synaptic vesicle cycling. J Clin Invest.

[29] Baker K., Gordon S.L., Melland H. (2018). SYT1-associated neurodevelopmental disorder: a case series. Brain.

[30] Melland H., Bumbak F., Kolesnik-Taylor A. (2022). Expanding the genotype and phenotype spectrum of SYT1-associated neurodevelopmental disorder. Genet Med.

[31] Bradberry M.M., Courtney N.A., Dominguez M.J. (2020). Molecular Basis for synaptotagmin-1-associated neurodevelopmental disorder. Neuron.

[32] Huang W., Yang Y., Che F., Wu H., Ma Y., Zhao Y. (2024). Lethal variant in the C2A domain may cause severe SYT1-associated neurodevelopmental disorder in the newborns. Neurogenetics.

[33] van Boven M.A., Mestroni M., Zwijnenburg P.J.G., Verhage M., Cornelisse L.N. (2024). A de novo missense mutation in synaptotagmin-1 associated with neurodevelopmental disorder desynchronizes neurotransmitter release. Mol Psychiatry.

[34] Melland H., Venter K., Leech S.L. (2023). Delineation of the pathogenic presynaptic mechanisms of synaptotagmin-1 variants. bioRxiv.

[35] Diril M.K., Wienisch M., Jung N., Klingauf J., Haucke V. (2006). Stonin 2 is an AP-2-dependent endocytic sorting adaptor for synaptotagmin internalization and recycling. Dev Cell.

[36] Sankaranarayanan S., De Angelis D., Rothman J.E., Ryan T.A. (2000). The use of pHluorins for optical measurements of presynaptic activity. Biophys J.

[37] Perin M.S., Johnston P.A., Ozcelik T., Jahn R., Francke U., Sudhof T.C. (1991). Structural and functional conservation of synaptotagmin (p65) in Drosophila and humans. J Biol Chem.

[38] Miura K. (2020). Bleach correction ImageJ plugin for compensating the photobleaching of time-lapse sequences. F1000Res.

[39] Lyles V., Zhao Y., Martin K.C. (2006). Synapse formation and mRNA localization in cultured Aplysia neurons. Neuron.

[40] Gordon S.L., Cousin M.A. (2013). X-linked intellectual disability-associated mutations in synaptophysin disrupt synaptobrevin II retrieval. J Neurosci.

[41] Landrum M.J., Lee J.M., Benson M. (2018). ClinVar: improving access to variant interpretations and supporting evidence. Nucleic Acids Res.

[42] Firth H.V., Richards S.M., Bevan A.P. (2009). DECIPHER: database of chromosomal imbalance and phenotype in humans using ensembl resources. Am J Hum Genet.

[43] Sobreira N., Schiettecatte F., Valle D., Hamosh A. (2015). GeneMatcher: a matching tool for connecting investigators with an interest in the same gene. Hum Mutat.

[44] Sparrow S.S., Balla R., Cicchetti D.V. (2016). Manual.

[45] Gray K., Tonge B., Einfeld S., Gruber C., Klein A. (2018).

[46] Ortibus E., Laenen A., Verhoeven J. (2011). Screening for cerebral visual impairment: value of a CVI questionnaire. Neuropediatrics.

[47] Rosenmund C., Stevens C.F. (1996). Definition of the readily releasable pool of vesicles at hippocampal synapses. Neuron.

[48] Dobrunz L.E., Stevens C.F. (1997). Heterogeneity of release probability, facilitation, and depletion at central synapses. Neuron.

[49] Stevens C.F., Williams J.H. (2007). Discharge of the readily releasable pool with action potentials at hippocampal synapses. J Neurophysiol.

[50] Zhou Q., Lai Y., Bacaj T. (2015). Architecture of the synaptotagmin-SNARE machinery for neuronal exocytosis. Nature.

[51] Cafiero C., Marangi G., Orteschi D. (2015). Novel de novo heterozygous loss-of-function variants in MED13L and further delineation of the MED13L haploinsufficiency syndrome. Eur J Hum Genet.

[52] Zhou A., Brewer K.D., Rizo J. (2013). Analysis of SNARE complex/synaptotagmin-1 interactions by one-dimensional NMR spectroscopy. Biochemistry.

[53] Wu Z., Dharan N., McDargh Z.A., Thiyagarajan S., O'Shaughnessy B., Karatekin E. (2021). The neuronal calcium sensor Synaptotagmin-1 and SNARE proteins cooperate to dilate fusion pores. Elife.

[54] Chang S., Trimbuch T., Rosenmund C. (2018). Synaptotagmin-1 drives synchronous Ca(2+)-triggered fusion by C(2)B-domain-mediated synaptic-vesicle-membrane attachment. Nat Neurosci.

[55] Pang Z.P., Shin O.H., Meyer A.C., Rosenmund C., Sudhof T.C. (2006). A gain-of-function mutation in synaptotagmin-1 reveals a critical role of Ca2+-dependent soluble N-ethylmaleimide-sensitive factor attachment protein receptor complex binding in synaptic exocytosis. J Neurosci.

[56] Rhee J.S., Li L.Y., Shin O.H. (2005). Augmenting neurotransmitter release by enhancing the apparent Ca2+ affinity of synaptotagmin 1. Proc Natl Acad Sci U S A.

[57] Kochubey O., Babai N., Schneggenburger R. (2016). A synaptotagmin isoform switch during the development of an identified CNS synapse. Neuron.

[58] Cooper A.P., Gillespie D.C. (2011). Synaptotagmins I and II in the developing rat auditory brainstem: synaptotagmin I is transiently expressed in glutamate-releasing immature inhibitory terminals. J Comp Neurol.

[59] Chicka M.C., Hui E., Liu H., Chapman E.R. (2008). Synaptotagmin arrests the SNARE complex before triggering fast, efficient membrane fusion in response to Ca2+. Nat Struct Mol Biol.

[60] Imig C., Min S.W., Krinner S. (2014). The morphological and molecular nature of synaptic vesicle priming at presynaptic active zones. Neuron.

[61] Yoshihara M., Littleton J.T. (2002). Synaptotagmin I functions as a calcium sensor to synchronize neurotransmitter release. Neuron.

[62] Huson V., Meijer M., Dekker R. (2020). Post-tetanic potentiation lowers the energy barrier for synaptic vesicle fusion independently of Synaptotagmin-1. Elife.

[63] Greif K.F., Asabere N., Lutz G.J., Gallo G. (2013). Synaptotagmin-1 promotes the formation of axonal filopodia and branches along the developing axons of forebrain neurons. Dev Neurobiol.

[64] Washbourne P., Thompson P.M., Carta M. (2002). Genetic ablation of the t-SNARE SNAP-25 distinguishes mechanisms of neuroexocytosis. Nat Neurosci.

[65] Xu W., Morishita W., Buckmaster P.S., Pang Z.P., Malenka R.C., Sudhof T.C. (2012). Distinct neuronal coding schemes in memory revealed by selective erasure of fast synchronous synaptic transmission. Neuron.

[66] Xu J., Mashimo T., Sudhof T.C. (2007). Synaptotagmin-1, -2, and -9: Ca(2+) sensors for fast release that specify distinct presynaptic properties in subsets of neurons. Neuron.

[67] Chen C., Arai I., Satterfield R., Young S.M., Jonas P. (2017). Synaptotagmin 2 is the fast Ca(2+) sensor at a central inhibitory synapse. Cell Rep.

[68] Bacaj T., Wu D., Yang X. (2013). Synaptotagmin-1 and synaptotagmin-7 trigger synchronous and asynchronous phases of neurotransmitter release. Neuron.

[69] Weber J.P., Toft-Bertelsen T.L., Mohrmann R., Delgado-Martinez I., Sorensen J.B. (2014). Synaptotagmin-7 is an asynchronous calcium sensor for synaptic transmission in neurons expressing SNAP-23. PLoS One.

[70] Banerjee A., Lee J., Nemcova P., Liu C., Kaeser P.S. (2020 Jun 3). Synaptotagmin-1 is the Ca2+ sensor for fast striatal dopamine release. Elife.

[71] Marqueze B., Boudier J.A., Mizuta M., Inagaki N., Seino S., Seagar M. (1995). Cellular localization of synaptotagmin I, II, and III mRNAs in the central nervous system and pituitary and adrenal glands of the rat. J Neurosci.

[72] Ullrich B., Li C., Zhang J.Z. (1994). Functional properties of multiple synaptotagmins in brain. Neuron.

[73] Turecek J., Regehr W.G. (2019). Neuronal regulation of fast synaptotagmin isoforms controls the relative contributions of synchronous and asynchronous release. Neuron.

