# Supplementary material for: Correlation between evoked neurotransmitter release and adaptive functions in SYT1-associated neurodevelopmental disorder
Source: eBioMedicine. 2024 Oct 30;109:105416. doi: 10.1016/j.ebiom.2024.105416 (PMC11564929; doi:10.1016/j.ebiom.2024.105416)
Supplement: Supplementary Figures and Tables [file mmc1.pdf]

Supplementary Material

Supplementary Table 1. Primers used for site-directed mutagenesis of SYT1-pH variants. Mutated bases in bold and underlined

SYT1 Variant (Human)	Primer	Sequence (5’-3’)
L159R	Forward<br>Reverse	CCAGAATAACCAGCG**G**TTGGTGGGAATCATC<br>GATGATTCCCACCAA**C**CGCTGGTTATTCTGG
T196K	Forward<br>Reverse	CCTGACAAAAAGAAGAAATTTGAGA**AG**AAAGTCCACCGGAAAACCC<br>GGGTTTTCCGGTGGACTTT**CT**TCTCAAATTTCTTCTTTTTGTCAGG
E209K	Forward<br>Reverse	CCCTCAATCCAGTCTTCAAT**A**AACAATTTACTTTCAAGGTACCCTA<br>CCGAGTAGGGTACCTTGAAAGTAAATTGTT**T**ATTGAAGACTGGATT
E219Q	Forward<br>Reverse	CAAGGTACCCTACTCG**C**AATTAGGTGGCAAAACCC<br>GGGTTTTGCCACCTAATT**G**CGAGTAGGGTACCTTG
M303V	Forward<br>Reverse	CCTGAAGAAG**G**TGGATGTGGGTGGCTTATCTG<br>CAGATAAGCCACCCACATCCA**C**CTTCTTCAGG
S309P	Forward<br>Reverse	GATGGATGTGGGTGGCTTA**C**CTGATCCCTACG<br>CGTAGGGATCAG**G**TAAGCCACCCACATCCATC
Y365C	Forward<br>Reverse	GGTGGTAACTGTTTTGGACT**G**TGACAAGATTGGCAAGAACG<br>CGTTCTTGCCAATCTTGTCA**C**AGTCCAAAACAGTTACCACC
G369D	Forward<br>Reverse	GGACTATGACAAGATTG**A**CAAGAACGACGCGATCGGC<br>GCCGATCGCGTCGTTCTTG**T**CAATCTTGTCATAGTCC

Supplementary Table 2a. Descriptive statistics for nerve terminal expression analysis (Fig 1C)

	n	Mean	SD	SEM	95% CI of mean		P-values (vs WT)*
WT	12	1.709	0.2486	0.07175	1.551	1.867	
L159R	8	0.9634	0.1599	0.05653	0.8297	1.097	**0.0076**
T196K	12	1.795	0.3795	0.1095	1.553	2.036	>0.9999
E209K	11	1.621	0.3594	0.1084	1.38	1.863	>0.9999
E219Q	11	1.939	0.4825	0.1455	1.615	2.263	>0.9999
M303V	11	1.856	0.4469	0.1347	1.556	2.156	>0.9999
S309P	10	1.935	0.4139	0.1309	1.639	2.231	>0.9999
Y365C	12	1.804	0.4498	0.1298	1.518	2.09	>0.9999
G369D	12	1.731	0.4680	0.1351	1.434	2.029	>0.9999
I368T	12	2.088	0.4311	0.1245	1.814	2.362	0.6015

**P-values for Kruskal-Wallis test with Dunn's multiple comparison test compared to WT.*

Supplementary Table 2b. Descriptive statistics for coefficient of variation analysis (Fig 2B)

	n	Mean	SD	SEM	95% CI of mean		P-values (vs WT)*
WT	9	46.44	4.676	1.559	42.84	50.03	
L159R	9	23.41	8.508	2.836	16.87	29.95	**<0.0001**
T196K	7	46.45	11.93	4.51	35.41	57.48	>0.9999
E209K	8	38.57	7.348	2.598	32.43	44.71	0.2691
E219Q	8	40.01	5.963	2.108	35.03	45	0.4936
M303V	8	40.36	11.44	4.045	30.8	49.93	0.5580
S309P	8	44.39	9.576	3.386	36.38	52.4	0.9987
Y365C	7	46.27	6.333	2.394	40.42	52.13	>0.9999
G369D	7	44.47	5.223	1.974	39.64	49.3	0.9993
I368T	7	46.19	5.939	2.245	40.7	51.68	>0.9999

**P-values for one-way ANOVA with Dunnett's multiple comparison test compared to WT.*

Supplementary Table 2c. Descriptive statistics for vesicular localisation analysis (Fig 2E)

	n	Mean	SD	SEM	95% CI of mean		P-values (vs WT)*
WT	9	54.04	6.927	2.309	48.72	59.37	
L159R	9	60.08	8.039	2.68	53.9	66.26	>0.9999
T196K	7	62.11	4.941	1.868	57.54	66.68	0.4231
E209K	8	60.3	6.496	2.297	54.87	65.73	>0.9999
E219Q	8	58.13	13.26	4.687	47.04	69.21	>0.9999
M303V	8	58.83	10.96	3.873	49.67	67.99	>0.9999
S309P	8	63.95	12.82	4.533	53.23	74.67	0.1454
Y365C	7	56.62	7.148	2.702	50.01	63.23	>0.9999
G369D	7	63.09	7.464	2.821	56.18	69.99	0.2279
I368T	7	65.22	7.465	2.822	58.31	72.12	0.0904

**P-values for Kruskal-Wallis test with Dunn's multiple comparison test compared to WT.*

Supplementary Table 2d. Descriptive statistics for C2B variant recycling pool size analysis (Fig 3A)

	n	Mean	SD	SEM	95% CI of mean		P-values (vs WT)*
WT	9	50.18	5.78	1.927	45.74	54.63	
M303V	8	37.51	7.263	2.568	31.44	43.58	**0.0143**
S309P	8	36.59	9.723	3.437	28.46	44.72	**0.0077**
Y365C	8	53.64	7.394	2.614	47.46	59.82	0.8692
G369D	9	49.12	9.068	3.023	42.15	56.09	0.9991
I368T	8	41.38	10.33	3.653	32.74	50.02	0.1361

**P-values for one-way ANOVA with Dunnett's multiple comparison test compared to WT.*

Supplementary Table 2e. Descriptive statistics for C2A variant recycling pool size analysis (Fig 3B)

	n	Mean	SD	SEM	95% CI of mean		P-values (vs WT)*
WT	10	53.88	8.94	2.827	47.48	60.27	
L159R	9	48.3	9.772	3.257	40.79	55.81	0.6012
T196K	11	57.44	6.779	2.044	52.88	61.99	0.8718
E209K	9	56.12	6.251	2.084	51.32	60.93	0.9826
E219Q	9	47.02	15.04	5.012	35.46	58.58	0.4059
I368T	7	50.3	8.752	3.308	42.2	58.39	0.9139

**P-values for one-way ANOVA with Dunnett's multiple comparison test compared to WT.*

Supplementary Table 2f. Descriptive statistics for C2B variant exocytosis tau analysis (Fig 3E)

	n	Mean	SD	SEM	95% CI of mean		P-values (vs WT)*
WT	9	23.45	3.412	1.137	20.82	26.07	
M303V	8	43.96	15.02	5.309	31.4	56.51	**0.0206**
S309P	8	57.49	25.72	9.093	35.99	78.99	**<0.0001**
Y365C	8	36.47	10.53	3.723	27.67	45.28	0.2322
G369D	9	35.14	11.82	3.94	26.05	44.23	0.2976
I368T	8	44.47	9.814	3.47	36.26	52.67	**0.0170**

**P-values for one-way ANOVA with Dunnett's multiple comparison test compared to WT.*

Supplementary Table 2g. Descriptive statistics for C2A variant exocytosis tau analysis (Fig 3F)

	n	Mean	SD	SEM	95% CI of mean		P-values (vs WT)*
WT	10	23.22	3.082	0.9747	21.02	25.43	
L159R	9	30.26	12.81	4.271	20.41	40.11	0.8385
T196K	11	27.6	8.145	2.456	22.13	33.07	>0.9999
E209K	9	30.61	8.24	2.747	24.28	36.95	0.2283
E219Q	9	37.01	17.24	5.747	23.76	50.27	0.0742
I368T	7	39.27	12.06	4.56	28.12	50.43	**0.0092**

**P-values for Kruskal-Wallis test with Dunn's multiple comparison test compared to WT.*

Supplementary Table 2h. Descriptive statistics for C2B variant initial exocytic rate analysis (Fig 4B)

	n	Mean	SD	SEM	95% CI of mean		P-values (vs WT)*
WT	9	0.0169	0.004124	0.001375	0.01373	0.02006	
M303V	8	0.007823	0.005722	0.002023	0.00304	0.01261	**0.0009**
S309P	8	0.005951	0.004176	0.001476	0.00246	0.009441	**<0.0001**
Y365C	8	0.01023	0.004702	0.001662	0.006301	0.01416	**0.0196**
G369D	9	0.01084	0.005058	0.001686	0.006953	0.01473	**0.0318**
I368T	8	0.006544	0.003324	0.001175	0.003765	0.009322	**0.0001**

**P-values for one-way ANOVA with Dunnett's multiple comparison test compared to WT.*

Supplementary Table 2i. Descriptive statistics for C2A variant initial exocytic rate analysis (Fig 4D)

	n	Mean	SD	SEM	95% CI of mean		P-values (vs WT)*
WT	10	0.02232	0.009249	0.002925	0.01571	0.02894	
L159R	9	0.01722	0.00646	0.002153	0.01225	0.02218	0.4016
T196K	11	0.0185	0.005556	0.001675	0.01476	0.02223	0.6250
E209K	9	0.01742	0.006819	0.002273	0.01218	0.02266	0.4403
E219Q	9	0.01551	0.008341	0.00278	0.009101	0.02192	0.1596
I368T	7	0.01009	0.004904	0.001854	0.005551	0.01462	**0.0048**

**P-values for one-way ANOVA with Dunnett's multiple comparison test compared to WT.*

Supplementary Table 2j. Descriptive statistics for C2B variant RRP size analysis (Fig 5B)

	n	Mean	SD	SEM	95% CI of mean		P-values (vs WT)*
WT	9	5.754	3.049	1.016	3.411	8.098	
M303V	11	3.148	1.63	0.4914	2.053	4.243	**0.0386**
S309P	9	2.274	2.072	0.6908	0.6805	3.867	**0.0053**
Y365C	9	3.81	1.965	0.6551	2.3	5.321	0.2107
G369D	10	4.487	2.464	0.7791	2.725	6.25	0.5712
I368T	9	1.978	1.37	0.4567	0.9252	3.031	**0.0022**

**P-values for one-way ANOVA with Dunnett's multiple comparison test compared to WT.*

Supplementary Table 2k. Descriptive statistics for C2A variant RRP size analysis (Fig 5D)

	n	Mean	SD	SEM	95% CI of mean		P-values (vs WT)*
WT	11	5.351	1.856	0.5597	4.104	6.599	
L159R	11	4.235	2.208	0.6658	2.751	5.718	0.6252
T196K	9	4.849	1.689	0.5631	3.551	6.148	0.9801
E209K	11	5.681	1.769	0.5333	4.492	6.869	0.9962
E219Q	11	6.304	3.26	0.9829	4.114	8.494	0.7510
I368T	8	1.806	0.974	0.3444	0.9922	2.621	**0.0034**

**P-values for one-way ANOVA with Dunnett's multiple comparison test compared to WT.*

Supplementary Table 3. SYT1-associated Neurodevelopmental Disorder participant characteristics

	N	Mean (SD)	Range	Median	Interquartile range
Sex	7 female<br>7 male	-			
Age[a]<br>*Years*	14	9.9 (6.17)	3.25 - 25.79	8.41	5.82 - 12.35
Global adaptive function<br>*Vineland Adaptive Behaviour Composite*	12	44.83 (17.98)	20 - 74	41	32.5 - 56
Behavioural and emotional difficulties<br>*DBC total problems T-score*	11	56.64 (8.21)	40 - 68	60	51.5 - 62.5

[a]Age at time of questionnaire completion (n=13) or clinical data collection (n=1)

Supplementary Table 4. SYT1-associated Neurodevelopmental Disorder participant clinical phenotypes

Clinical feature<br>HPO Term Identifier[a]	Data available (n)	Frequency of Feature (n)	Frequency of Feature (%)	Subtype (n)
Delayed speech and language development<br>HP: 0000750	14	14	100	Mild = using words and phrases (5); moderate = using single words only (2); severe = not using any words (4); unable to classify as under age 5 years or insufficient information (3)
Abnormal eye physiology<br>HP: 0012373	14	12	86	Strabismus (7); nystagmus (4); hypermetropia (1); visual impairment unspecified (1)
Motor delay<br>HP: 0001270	14	14	100	Mild = walked by 3 years (5); moderate = walked by 5 years (2); severe = walked after 5 years or nonambulatory over the age of 5 (5); unable to classify because nonambulatory under the age of 5 years (2)
Neonatal hypotonia<br>HP: 0001319	14	11	79	-
Abnormality of movement<br>HP: 0100022	14	11	79	Dystonia (5); chorea (7); dyskinesia (3); ataxia (5); myoclonus (3); tremor (3); stereotypies (5); Tourette syndrome (1)
Sleep disturbance<br>HP: 0002360	13	9	69	-
Abdominal symptom<br>HP: 0011458	14	8	57	Feeding difficulties (6); gastroesophageal reflux (2); drooling (1); constipation (2); urinary infection (2); pancreatitis with pseudocysts (1)
Self-injurious behaviour<br>HP: 0100716	14	11	79	Finger biting or chewing (7); head banging (2); skin picking (1); hair pulling (1); other or unspecified (2)
Seizure<br>HP: 0001250	14	4	29	-

MRI abnormality HP: 0012639	13	3	23	-
Abnormality of the respiratory system HP: 0002086	13	2	15	Hyperventilation with cyanosis (1); autonomic dysfunction with hypotension (1)
Phenotypic abnormality HP: 0000118	13	4	31	Undescended testicle (1); dermoid cyst (1); talipes (2)
Abnormality of prenatal development or birth HP: 0001197	14	2	14	Mild prematurity (1); neonatal resuscitation (2)

[a]Human Phenotype Ontology (https://hpo.jax.org/app/).

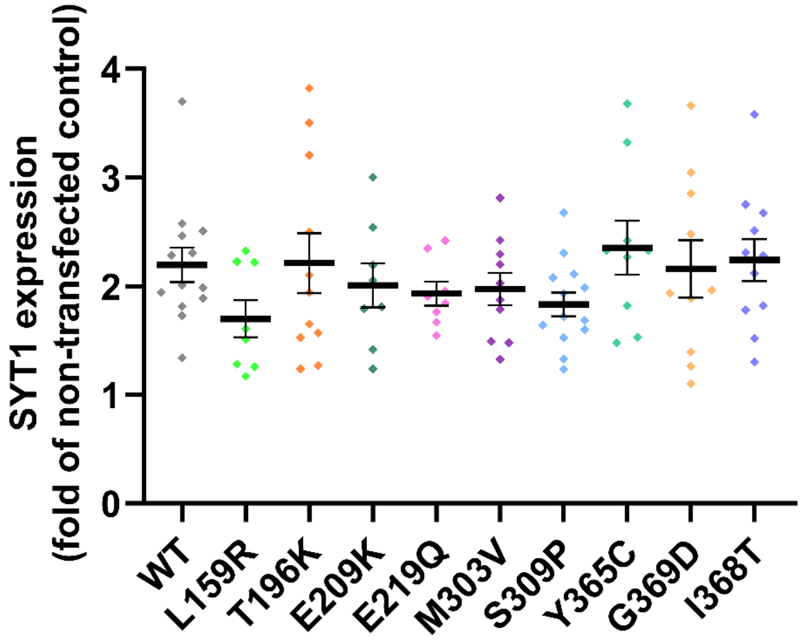

Supplementary Figure 1. SYT1 variants are expressed as efficiently as the WT protein within neuronal cell soma. Hippocampal neurons co-transfected with SYT1-pHluorin variants and empty vector pcDNA3.1 were fixed and immunolabelled for GFP (i.e. pHluorin) and SYT1. SYT1 somatic expression levels are expressed as the SYT1 immunofluorescence intensity in transfected neurons, relative to non-transfected neurons in the same field of view. Data displayed as mean ± SEM, n = 8-13. One-way ANOVA with Dunnett's multiple comparison test compared to WT (n = 13); L159R p = 0.42 (8), T196K p > 0.99 (11), E209K p = 0.99 (8), E219Q p = 0.95 (8), M303V p = 0.97 (10), S309P p = 0.63 (13), Y365C p = 0.99 (9), G369D p > 0.99 (10), I368T p > 0.99 (11). For all experiments, 'n' refers to an individual field of view from an independent coverslip. All experiments were repeated across at least 3 independent cultures, with each culture comprising at least 3 embryos.

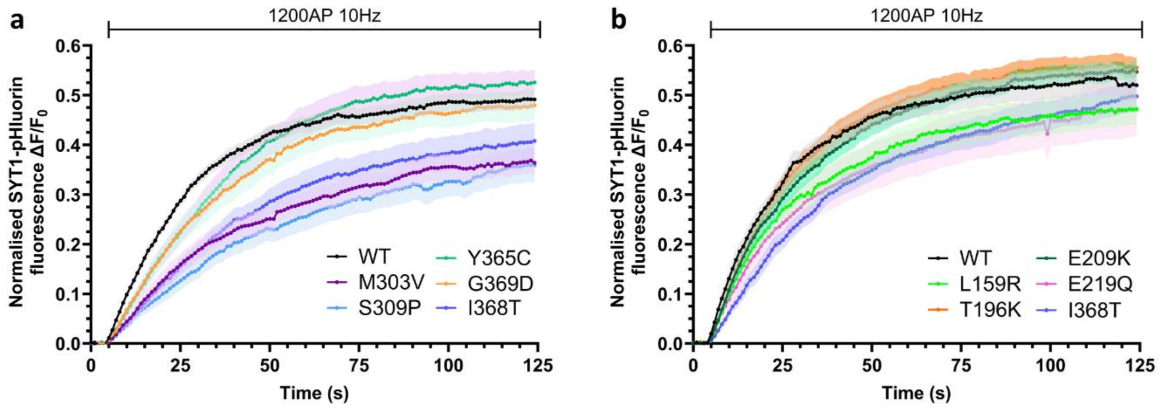

Supplementary Figure 2. C2B and C2A domain SYT1 variants induce a dominant-negative slowing of exocytosis. Hippocampal neurons transfected with SYT1-pHluorin variants or wild-type SYT1-pHluorin were stimulated with 1200 AP at 10 Hz in the presence of bafilomycin A1, and then perfused with ammonia buffer to reveal total SYT1-pHluorin fluorescence. (**a, b**) Time course of mean $\Delta F/F_0$ of C2B (A) or C2A (B) domain SYT1-pHluorin variants normalised to peak amplitude of fluorescence change induced by ammonia buffer perfusion. Shaded areas indicate the standard error of the mean (SEM).

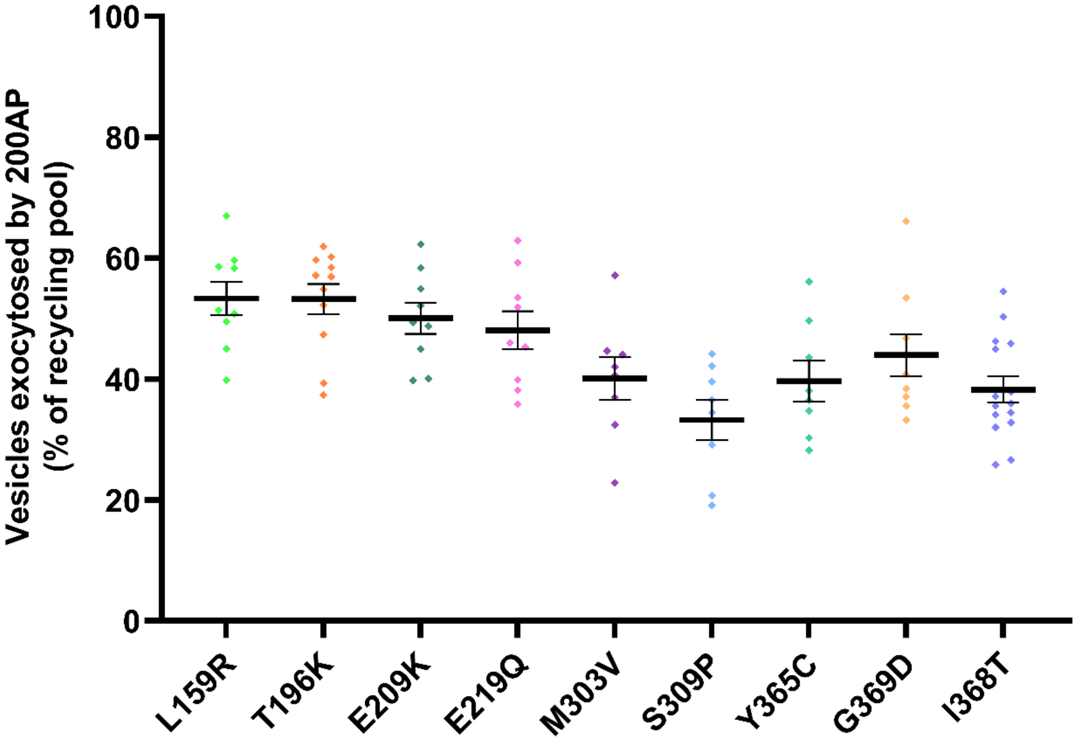

Supplementary Figure 3. The number of synaptic vesicles exocytosed cumulatively by 200 AP stimulation. The percentage of synaptic vesicles cumulatively exocytosed following 200AP (i.e. after 20 seconds of stimulation) stimulation, as a proportion of the total recycling pool of vesicles, was determined for all SYT1 variants. Data displayed is mean +/- SEM.

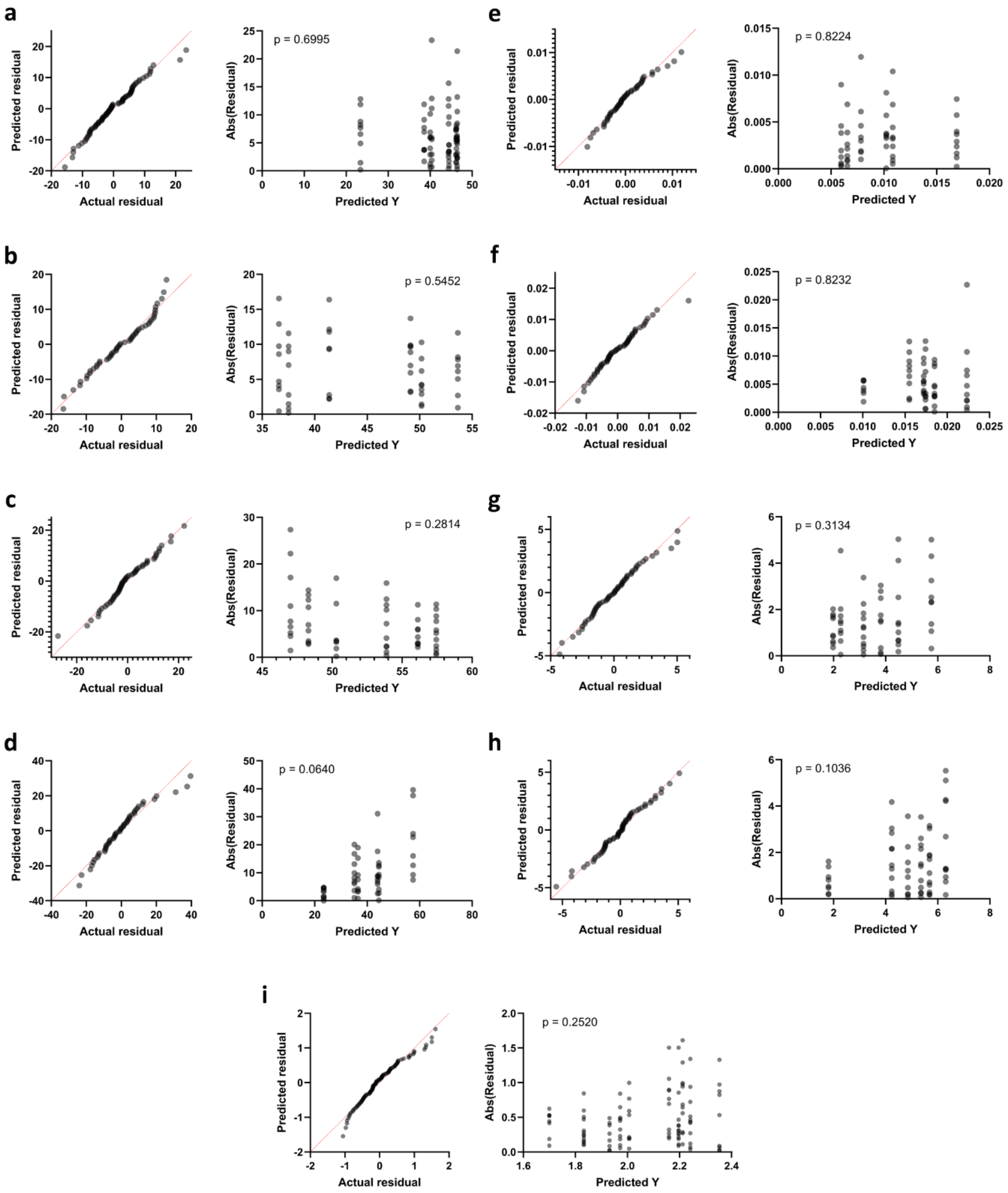

Supplementary Figure 4. Testing of assumptions for one-way ANOVA data. Q-Q residual plots (*left panel*) for assessing normality of data, and homoscedasticity plots (*right panel*) for assessing homogeneity of variance between data groups, for data sets analysed through one-way ANOVA which include (**a**) coefficient of variation (CV), Fig 2b, (**b**) C2B variant recycling pool (RP) size, Fig 3a, (**c**) C2A variant RP size, Fig 3b, (**d**) C2B variant exocytic tau, Fig 3e, (**e**) C2B variant initial exocytic rate, Fig 4b, (**f**) C2A variant initial exocytic rate, Fig 4d, (**g**) C2B variant readily releasable pool (RRP) size, Fig 5b, (**h**) C2A variant RRP size, Fig 5d, and (**i**) somatic SYT1 expression levels, Supp Fig 1. Brown-Forsythe test for homoscedasticity was performed for each data set, with the corresponding p-values reported.

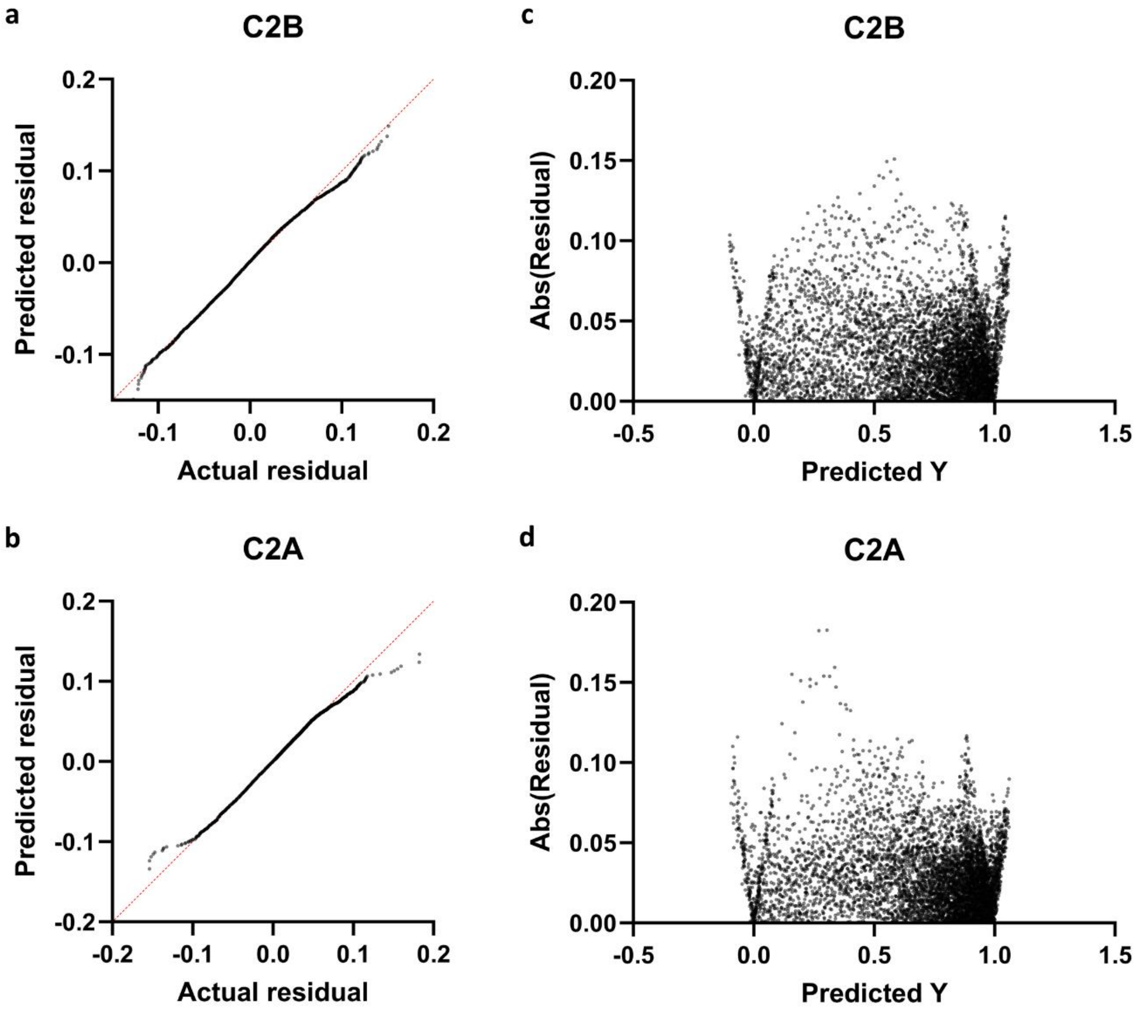

Supplementary Figure 5. Testing of assumptions for repeated measures (two-way) ANOVA data. (**a, b**) Q-Q residual plots of data sets analysed through repeated measures ANOVA, for assessing normality of data. (**c, d**) Homoscedasticity plots of data sets analysed through repeated measures ANOVA, for assessing homogeneity of variance between data groups. Repeated measures mixed model ANOVA was performed with no assumption of sphericity and using the Geisser-Greenhouse correction.

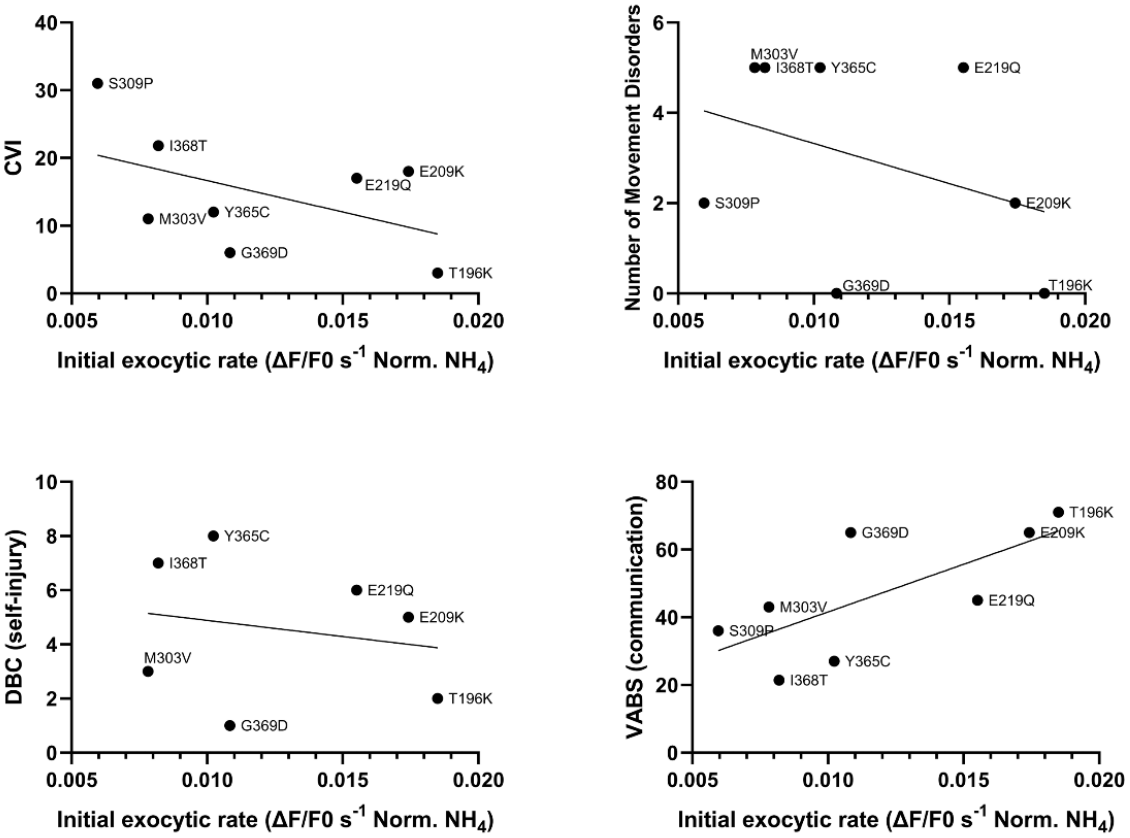

Supplementary Figure 6. Scatter plots of non-significant correlations between initial exocytic rate in the presence of SYT1 variants and clinical measures of individuals with SYT1 variants. Quantitative phenotypes exhibited by individuals harbouring SYT1 variants were each correlated with the functional measure of initial exocytic rate over the first 5 seconds of 10Hz stimulation. Clinical measures include the communication subscale of the Vineland Adaptive Behaviour Scale (VABS), number of movement disorders, self-injury scores from Developmental Behaviour Checklist (DBC), and cerebral visual impairment (CVI). Correlation values can be found in Table 1 of the main text.

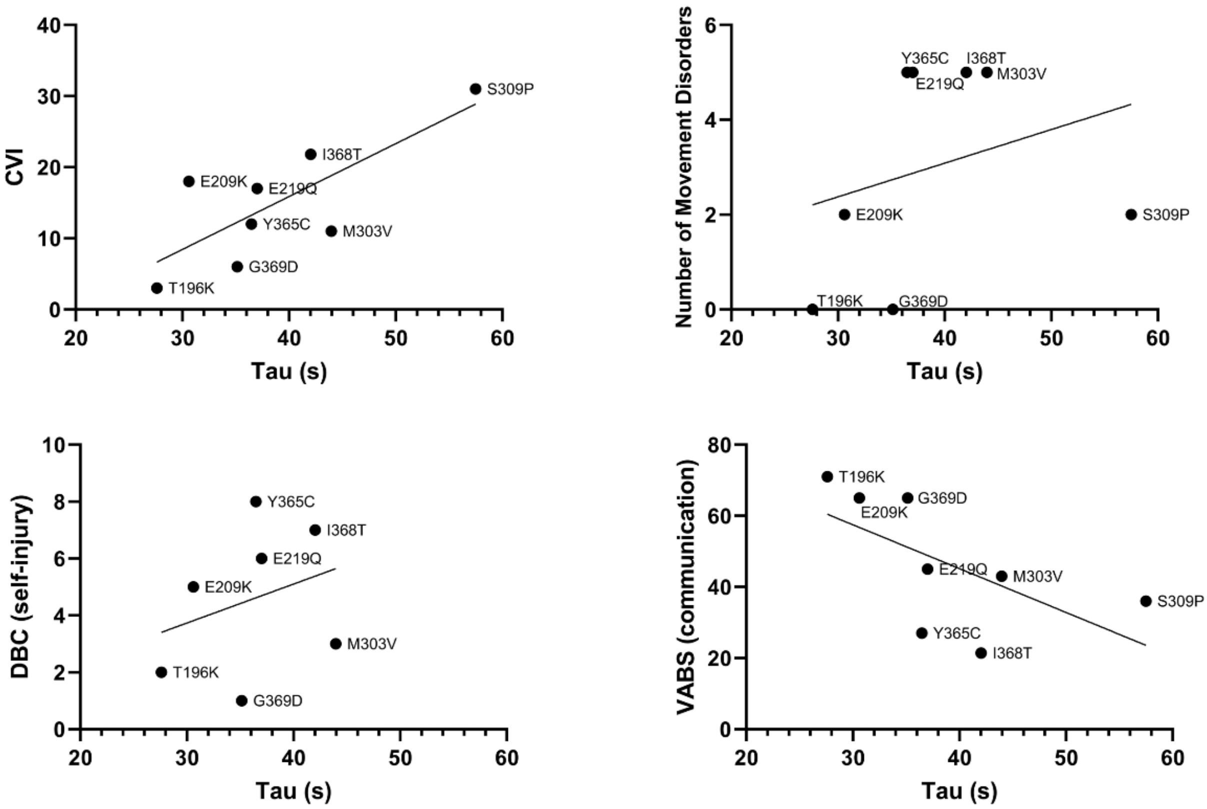

Supplementary Figure 7. Scatter plots of non-significant correlations between tau constant values in the presence of SYT1 variants and clinical measures of individuals with SYT1 variants. Quantitative phenotypes exhibited by individuals harbouring SYT1 variants were each correlated with the functional measure for overall exocytic rate (tau). Clinical measures include the communication subscale of the Vineland Adaptive Behaviour Scale (VABS), number of movement disorders, self-injury scores from Developmental Behaviour Checklist (DBC), and cerebral visual impairment (CVI). Correlation values can be found in Table 1 of the main text.

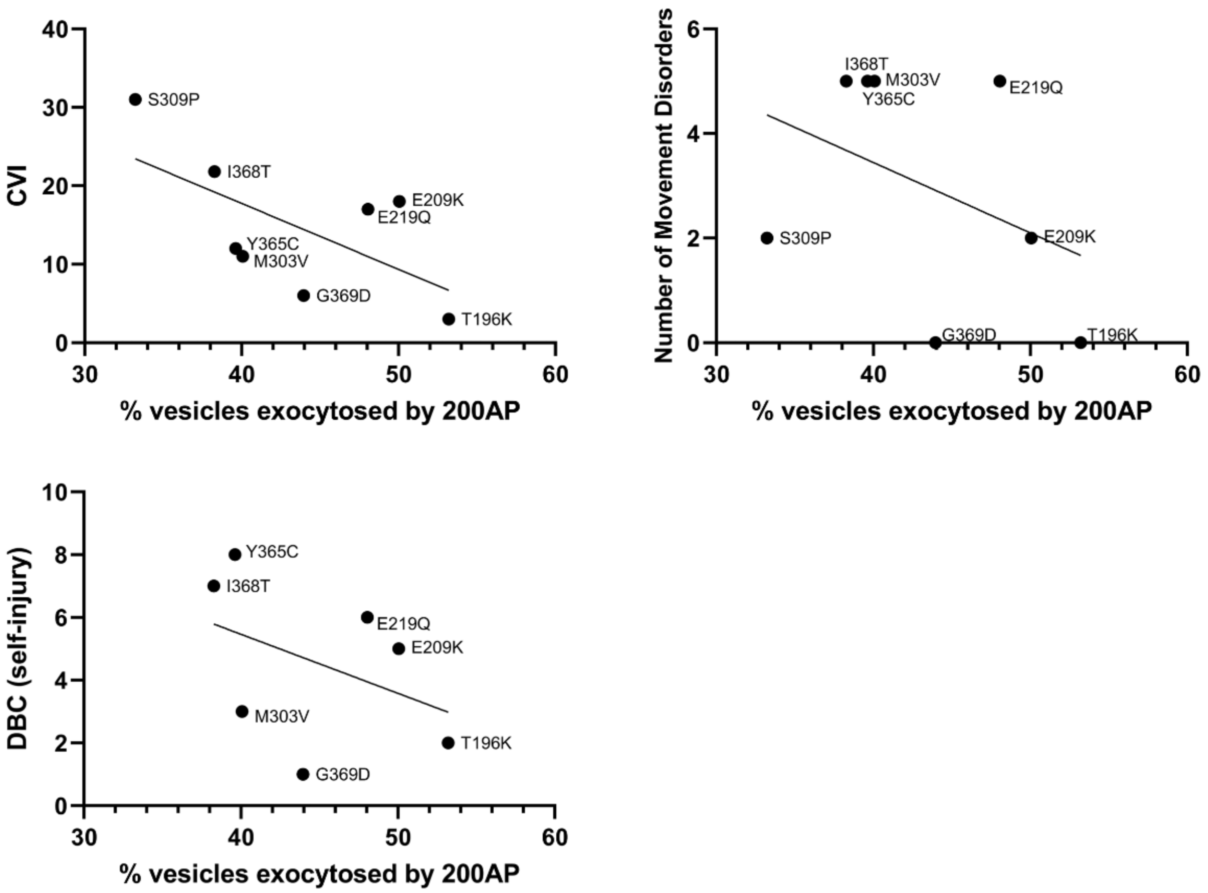

Supplementary Figure 8. Scatter plots of non-significant correlations between the percentage of versicles fused by 200AP in the presence of SYT1 variants and clinical measures of individuals with SYT1 variants. Quantitative phenotypes exhibited by individuals harbouring SYT1 variants were each correlated with the functional measure of percentage of vesicles fused by 200AP. Clinical measures include number of movement disorders, self-injury scores from Developmental Behaviour Checklist (DBC), and cerebral visual impairment (CVI). Correlation values can be found in Table 1 of the main text.
